# Linkage Between Dissolved Organic Matter Transformation, Bacterial Carbon Production, and Diversity in a Shallow Oligotrophic Aquifer: Results From Flow-Through Sediment Microcosm Experiments

**DOI:** 10.3389/fmicb.2020.543567

**Published:** 2020-11-05

**Authors:** Roland Hofmann, Jenny Uhl, Norbert Hertkorn, Christian Griebler

**Affiliations:** ^1^Institute of Groundwater Ecology, Helmholtz Center Munich, Neuherberg, Germany; ^2^Research Unit Analytical Biogeochemistry, Helmholtz Center Munich, Neuherberg, Germany; ^3^Division of Limnology, Department of Functional and Evolutionary Ecology, University of Vienna, Vienna, Austria

**Keywords:** groundwater, oligotrophy, bacterial production, dissolved organic matter, mass spectrometry, carbon cycling, carbon use efficiency, microbial activity

## Abstract

Aquifers are important reservoirs for organic carbon. A fundamental understanding of the role of groundwater ecosystems in carbon cycling, however, is still missing. Using sediment flow-through microcosms, long-term (171d) experiments were conducted to test two scenarios. First, aquifer sediment microbial communities received dissolved organic matter (DOM) at low concentration and typical to groundwater in terms of composition (DOM-1x). Second, sediments received an elevated concentration of DOM originating from soil (DOM-5x). Changes in DOM composition were analyzed *via* NMR and Fourier transform ion cyclotron resonance mass spectrometry (FT-ICR-MS). Carbon production, physiological adaptations and biodiversity of groundwater, and sediment prokaryotic communities were monitored by total cell counts, substrate use arrays, and deep amplicon sequencing. The experiments showed that groundwater microbial communities do not react very fast to the sudden availability of labile organic carbon from soil in terms of carbon degradation and biomass production. It took days to weeks for incoming DOM being efficiently degraded and pronounced cell production occurred. Once conditioned, the DOM-1x supplied sediments mineralized 294(±230) μgC L^−1^_sed_ d^−1^, 10-times less than the DOM-5x fed sediment communities [2.9(±1.1) mgC L^−1^_sed_ d^−1^]. However, the overall biomass carbon production was hardly different in the two treatments with 13.7(±4.8) μgC L^−1^_sed_ d^−1^ and 14.3(±3.5) μgC L^−1^_sed_ d^−1^, respectively, hinting at a significantly lower carbon use efficiency with higher DOM availability. However, the molecularly more diverse DOM from soil fostered a higher bacterial diversity. Taking the irregular inputs of labile DOM into account, shallow aquifers are assumed to have a low resilience. Lacking a highly active and responsive microbial community, oligotrophic aquifers are at high risk of contamination with organic chemicals.

## Introduction

The water saturated terrestrial subsurface harbors the quantitatively most extensive freshwater ecosystems. Near ~100 times more freshwater occurs in the terrestrial subsurface than in surface waters (Danielopol et al., 2003). However, with the exclusion of light as energy source and an obviously minor contribution of chemoautotrophy, the predominantly heterotrophic communities in shallow aquifers are highly dependent on organic matter originating from the surface, received *via* groundwater recharge (Pabich et al., 2001; Goldscheider et al., 2006; Shen et al., 2015). While our knowledge on carbon cycling is well developed for surface waters (e.g., Drake et al., 2017; Tranvik et al., 2018), we still lack a detailed understanding of the fate of organic matter and its linkage to the heterotrophic production for groundwater ecosystems. Moreover, available knowledge almost exclusively corresponds to the groundwater component ignoring dissolved organic matter (DOM) and microbial communities attached to the sediment and/or rock matrix (Griebler et al., 2014; Hofmann and Griebler, 2018).

Groundwater is recharged from precipitation and seepage or surface water exfiltration into the subsurface. On its way down into the aquifer, seepage water is typically depleted in dissolved organic carbon (DOC) in terms of quantity and quality (Pabich et al., 2001; Lennon and Pfaff, 2005; Shen et al., 2015). In consequence, groundwater ecosystems are typically poor in organic carbon and energy (Aiken, 2002; Regan et al., 2017). However, at times of heavy rain and floods, shallow aquifers may receive irregular pulses of DOM (Jenerette et al., 2008; Peter et al., 2012a; Vaughan et al., 2017). Moreover, while there is plenty data on DOC concentration dynamics (e.g., Goni and Gardner, 2003; Strohmeier et al., 2013), only a few studies also addressed its change in composition and bioavailability with distance to surface and time (Einsiedl et al., 2007; Longnecker and Kujawinski, 2011; Peter et al., 2012b; Shabarova et al., 2014; Shen et al., 2015; Pracht et al., 2018; Wu et al., 2018). Even more important, the link between dynamics in DOM concentration and composition and heterotrophic production as well as microbial biodiversity remains largely unexplored.

It is well accepted that climate change is related to severe changes in global carbon cycling (Ciais et al., 2014). We lack detailed information on quantitative and mechanistic aspects of organic carbon turnover, sequestration, and release in groundwater ecosystems (Downing and Striegl, 2018), which is mainly governed by microorganisms. To better understand the carbon cycle in shallow groundwater, we thus need a first clue of the time and efficiency it is turned over, including a detailed understanding of its dynamics and molecular transformations, and partitioning into new microbial biomass and CO_2_. The few studies conducted indicate comparably low microbial carbon use efficiencies in oligotrophic aquifers (Thorn and Ventullo, 1988; Wilhartitz et al., 2009; Fasching et al., 2014; Hofmann and Griebler, 2018). This may explain, in combination with the comparable low DOC concentration and bioavailability, the rather low microbial biomass and activity present in oligotrophic aquifers, which is 1–4 orders of magnitude lower than in surface waters (Pedersen, 2000; Griebler and Lueders, 2009). Increased microbial biomass and activity is found in transition zones, i.e., the interface of the saturated and unsaturated zones as well as in the hyporheic zone (Madsen and Ghiorse, 1993; Griebler and Lueders, 2009; Stegen et al., 2015), or zones of organic contamination (Anneser et al., 2008, 2010; Herzyk et al., 2017).

In our study, we monitored microbial growth for groundwater and sediment communities in flow-through sediment microcosms, simulating a shallow sandy aquifer, for a period of 171 days. The model aquifer systems received a continuous supply of DOM, on one hand at a concentration and composition typical for oligotrophic groundwater (1.6–1.9 mg L^−1^) and, on the other hand, typical for a DOM pulse from forested land to the shallow aquifer (5.1–8.4 mg L^−1^) as a result of a heavy rain event, i.e., DOM at elevated concentrations and bioavailability. In detail, we examined changes in concentrations and composition of DOM when passing through the model aquifer by means of Fourier transform ion cyclotron resonance mass spectrometry (FT-ICR-MS), NMR, and DOC analysis. Depletion of individual DOM components was related to adsorption and microbial consumption and transformation. Microbial (prokaryotic) biomass and growth was monitored *via* flow cytometric counts of freely suspended and attached cells. Microbial activity and carbon production were evaluated by measuring intracellular ATP and carbon balancing. The immediate capability and short-term metabolic flexibility of microbes to degrade different classes of organic compounds were tested, running BIOLOG Phenotype MicroArray Carbon source plates. Finally, we looked into temporal changes in composition of the sediment bacterial communities exposed to low and high DOM supply by means of 454 pyrosequencing.

## Materials and Methods

### 2D-Aquifer Microcosms

**Figure 1:**
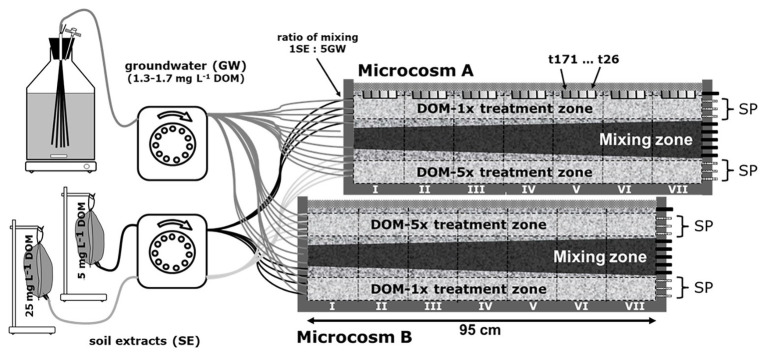
Experimental setup of the 2D microcosms. Two microcosms were run in parallel. Both microcosms were supplied with feeding solution DOM-1x, DOM-5x, and groundwater. In microcosm A, DOM-1x was infiltrated to the top zone, groundwater to the middle zone, and DOM-5x to the bottom zone. In microcosm B, the order was in reverse. DOM-1x and DOM-5x were supplied through four adjacent inlet ports at the bottom and top of the microcosm, separated by three ports receiving only groundwater. DOM-1x and DOM-5x were composed from soil extract (low and high conc.) and groundwater mixed 5:1 at the inlets to the microcosm. For water sampling, only the outflow of the three top and bottom ports (sampling ports; SP) was used. The two sediment zones receiving extra dissolved organic matter (DOM) and traversing each of the microcosms in a longitudinal direction are termed “treatment zones” (horizontal dashed lines). Vertical sediment sections are termed segments (segments I–VII, vertical black lines). Sediment was collected regularly in tanks A and B from the top (location is highlighted by the gray bars in microcosm A).

Two 2D-flow-through microcosms (L: 0.95 × W: 0.01 × H: 0.13 m) were packed with freshly sampled aquifer sediment, wet sieved with groundwater for the fine to medium sand fraction (200–2,000 μm). The aquifer sand originated from a gravel pit near Munich, Germany (Rühle et al., 2013). For the establishment of a stable microbial community, the sediment was infiltrated with pristine, quaternary groundwater from a shallow, porous aquifer in Neuherberg (Germany) for more than a year before it was packed into the microcosms. Sediment packing was performed, avoiding inclusion of gas bubbles as described in Bauer et al. (2009). Peristaltic pumps (injection volume 58 μl min^−1^ port^−1^, Ismatec, Germany), equipped with Fluran tubing (1.02 mm ID, Ismatec, Germany), were used to maintain a constant groundwater inflow into the microcosms. To prevent changes in flow rates, tubings were changed once a week. At the outlet of the microcosm, another peristaltic pump was connected with a slightly higher pumping rate to maintain a small unsaturated zone and a constant flow field (Hofmann et al., 2016; Figure 1).

Prior to the supply with the two feeding solutions, DOM-1x and DOM-5x, the sediment microcosms were infiltrated and conditioned with natural groundwater for 2 days. In consideration of the water retention time in the microcosm (14.8 h on average), sampling of sediments and outlet water took place with a delay of 1 day to the collection of inlet water. Prior to the supply of any extra DOM, our monitoring started collecting reference samples (t0) at the microcosms’ outlet that consisted of non-treated groundwater after it had passed the sediment. Time point 1 (t1) refers to the first samples at the outlet that contained feeding solutions, collected 24 h after the supply with DOM-1x and DOM-5x started.

Two microcosms were run in parallel, however, with a slightly different setup. Both microcosms had in common the supply with feeding solution one (DOM-1x), feeding solution two (DOM-5x), and natural groundwater. While in microcosm A, DOM-1x was infiltrated in the top zone, natural groundwater in the middle zone, and DOM-5x in the bottom zone, this order was in reverse in microcosm B (Figure 1). The feeding solutions (DOM-1x and DOM-5x) were infiltrated through four adjacent inlet ports at the bottom and top of the microcosm, separated by three ports receiving only natural groundwater. According to tracer results, the zones sampled were not affected by the other treatments. As a consequence of this pseudo-replication design, sediment receiving DOM-1x was available for sampling in microcosm A, and sediment receiving DOM-5x in microcosm B. The microcosms were run at 14°C. Feeding solutions DOM-1x and DOM-5x were spiked with the conservative tracers LiCl (f. conc. 25 mg L^−1^) and KBr (f. conc. 25 mg L^−1^). After passage of the feeding solutions through the microcosm, only the outflow of the three saturated top and bottom ports (termed treatment zones; Figure 1) was used for analysis. Due to transversal dispersion, ports 5–9 were affected by hydrodynamic mixing and not considered for further analysis. For better distinction, areas traversing the setup were divided into “treatment zones” (horizontal black lines) and vertical areas as “segments” (segments I–VII, vertical black lines). The gray areas in the upper microcosm (Figure 1) point at spots where sediment samples were collected.

Since the DOM feeding solutions had to be replaced once after 46 days, the results, wherever appropriate, are reported individually for the two experimental phases characterized by slightly different DOM input (Phase 1: t1–t46, Phase 2: t77–t171).

### Groundwater and DOM Supplies

Natural groundwater was supplied on-line from the local shallow Quaternary sandy-gravely aquifer. The DOM stock solution was prepared by extraction of natural DOM from O and A horizons of a conifer forest soil from the area of Munich, Germany. Soil was mixed with ultrapure water (25% w/v) and shaken for 2 days at 12°C in the dark. Larger particles were then removed by sedimentation and centrifugation, followed by filtration (0.22 μm, PVF, Millipore, United States) of the supernatant. The resulting DOM stock solution was stored in the dark at 4°C until further usage.

The two differently concentrated DOM feeding solutions were prepared by autoclaving of a sufficient amount of groundwater under N_2_ atmosphere. Subsequently, the anoxic groundwater was mixed with aliquots from the DOM stock solution to receive a final DOC concentration of 5 and 25 mg L^−1^, respectively. LiCl or KBr was amended as conservative tracers as mentioned above. Biogon gas (80% N_2_, 20% CO_2_) was used to transfer the DOM solutions into sterile Tedlar bags (Restek, United States) and kept under hypoxic/anoxic conditions at 12°C during supply to the microcosms. Before entering the microcosms, the two different DOM solutions were combined with well oxygenated groundwater at a ratio of 1:5 to obtain the feeding solutions termed DOM-1x and DOM-5x.

### Sampling of the Microcosm

Inflow water samples were collected by briefly disconnecting capillaries from the inlet ports of selected DOM-1x and DOM-5x zones. Water samples at the outlet were collected, with a delay of 1 day, at the microcosms outlets from each or selected ports into sterile falcon tubes at t0, t1, t7, t28, t46, t77, t97, t126, and t171.

To follow spatiotemporal dynamics within the microcosms, each microcosm was subdivided into seven segments [I (close to inlet) to VII (close to outlet), 13 cm in length each]. Sediment samples were obtained from the uppermost layer. Collection of sediments always started from the side of the outflow and ended at the inflow side. Similarly, to avoid sampling at already earlier disturbed points, collection of sediment within the individual sectors started at the side closer to the outlet (e.g., t0) and ended at the side closer to the inlet (t171). Small sediment cores of 3 cm depth using a sterile 3 ml-syringe with the head cut off were taken. Subsequently, the spot was immediately refilled with fresh, conditioned sediment. The upper 1.5 cm of the sediment cores collected were discarded, and the remaining sediment dedicated to different analyses. Sediment samples were collected before the supply of feeding solutions (t0) and at t46, t77, t126, and t171.

### Physical-Chemical Variables

For the analysis of major ions, water samples were filtered through a 0.2 μm syringe filter (PVF, Millipore, United States) and 200 μl of filtrate measured in an ion chromatograph (Dionex ICS-1100; Thermo Fisher Scientific, United States) as described before (Anneser et al., 2008).

DOC concentration in water samples was determined after filtration (0.45 μm, PVF, Millipore, United States) and acidification with HCl to a pH of <2 as non-purgeable dissolved carbon with a TOC analyzer (Shimadzu TOC 5000A).

### DOM Mineralization

Mineralization of organic carbon was calculated from the import and export of DOC for the respective treatment zones DOM-1x and DOM-5x in the microcosms. Only the period t28–t171 was considered, ignoring initial phases of DOM sorption and acclimatization of microbial communities. The mineralization rates may thus be understood as an “optimum” rate. Moreover, we did not consider in our calculations the carbon assimilated in microbial biomass, which was less than 5% in the DOM-1x treatment and less than <0.5% in the DOM-5x treatment.

### DOM Composition

#### Fourier Transform Ion Cyclotron Resonance Mass Spectrometry

Around 250 ml of water samples were filtered through GF/F filters (Whatman, GE Healthcare) and further acidified using 32% hydrochloric acid (Merck, Germany) to pH 2. Each sample was extracted using Bond Elut PPL solid phase cartridges (100 mg, 1 ml; Agilent Technologies) following a protocol already described by Dittmar et al. (2008) and the methanolic solid phase extracts (SPE-DOM) were diluted 1:20 in pure methanol (Hypergrade LC-MS, Merck, Germany) prior to analysis by FT-ICR-MS.

Ultrahigh resolution FT-ICR mass spectra were acquired using a 12T Bruker SolariX mass spectrometer (Bruker Daltonik, Bremen, Germany) equipped with an Apollo II electrospray ionization (ESI) source in negative mode. SPE-DOM samples were directly injected into the ESI source with a flow rate of 120 μl h^−1^ at a nebulizer gas pressure of 220 kPa and a dry gas flow rate of 4 L min^−1^. The source temperature of 200°C was maintained to ensure rapid desolvatation in the ionized droplets. The spectra were acquired in the mass range of 150–1,000 m/z with a time domain of four megawords and 300 scans were accumulated for each spectrum. Spectra were first externally calibrated on clusters of a standard arginine solution and internal calibration was systematically done in the presence of natural organic matter reaching accuracy values lower than 1 ppm.

Calculation of elemental formulas for each peak was done in a batch mode by an in-house written software tool (Tziotis et al., 2011). The generated formulae were validated by setting sensible chemical constraints [N rule, O/C ratio ≤ 1, H/C ratio ≤ 2n + 2 (C_n_H_2n+2_), with element counts C ≤ 100, H ≤ 200, O ≤ 80, N ≤ 3, S ≤ 2 and mass accuracy window (set at ±0.5 ppm)]. Final formulae were generated and categorized into groups containing CHO, CHNO, CHOS, or CHNOS molecular compositions, which were used to reconstruct the group-selective mass spectra (Schmitt-Kopplin et al., 2010). The computed average values for H, C, N, O, and S (atom %) and the H/C and O/C ratios were based upon intensity-weighted averages of mass peaks with assigned molecular formulae, which comprised ~50% of observed mass peaks.

#### Nuclear Magnetic Resonance Spectroscopy

^1^H NMR detected spectra of SPE-DOM were acquired with a Bruker Avance NMR spectrometer at 800.13 MHz (*B*_0_ = 18.7 T) at 283 K. Around 200 μg (2 mg for forest soil extract) of solid, obtained by evaporation of SPE-DOM samples, was dissolved in ~50 μl CD_3_OD (Merck. 99.95% ^2^H) solution and analyzed with a 5 mm z-gradient ^1^H/^13^C/^15^N/^31^P QCI cryogenic probe (90° excitation pulses: ^13^C ~^1^H ~10 μs) in sealed 1.7 mm Bruker MATCH tubes (forest soil: 150 μl CD_3_OD, 3 mm MATCH tube). Acquisition conditions were identical to those described in Hertkorn et al. (2013). The number of scans ranges from 272 (forest soil leachate) to 4,544 (groundwater inflow) in 1D ^1^H NMR spectra (Bruker pulse sequence noesypr1d); further NMR acquisition conditions are given in Supplementary Table S2.

### Community Substrate Usage

The metabolic diversity of the microbial community with respect to the usage of multiple substrates was assessed using Phenotype MicroArray Plates 1 and 2 (Biolog, United States; Low-Décarie et al., 2015) for water samples collected from DOM-1x and DOM-5x fed sediment at t171. The wells were loaded with 200 μl water sample and incubated for 25 days at 12°C in the dark. Changes in absorbance were evaluated once a day in a Plate reader (Victor 3, Perkin Elmer, United States) at a wavelength of 595 nm. According to their chemical properties, substrates were grouped into six classes (amines/amides, amino acids, carbohydrates, carboxylic acids, miscellaneous, and polymers; Preston-Mafham et al., 2002). Differences in substrate usage were analyzed by comparing the number of substrates of a group metabolized at different time points over the course of the incubation.

### Microbiological Biomass and Activity

Total cell counts (TCC) of pretreated samples (100 μl sediment or 500 μl water) were determined after staining with SybrGreen I following the protocol of Bayer et al. (2016).

Total ATP in water samples was determined following a protocol of Hammes et al. (2010) with only slight modifications. In detail, 1 ml of water sample (triplicates) was warmed to 38°C and mixed with 50 μl of BacTiter-Glo solution (Promega, Germany). After 1 min of incubation with reagent, luminescence was directly measured by integrating 10 individual measurements, one each second, on a GloMax 20/20 Luminometer (Promega, Germany). Total ATP in sediment samples was determined after warming 200 μl portions of sediment (triplicates) to 38°C and shaking (900 rpm) for 15 min, followed by mixing with 100 μl of BacTiter-Glo reagent (Promega, Germany) and incubation for 2.5 min. Next, the mixture was diluted with 900 μl preheated (38°C) ultrapure water (Thermo Fisher, United States), briefly mixed and centrifuged for 10 s (25,000 *g*). The supernatant was then transferred into a fresh, autoclaved Eppendorf tube for immediate direct bioluminescence measurement. Calibration was performed by determining the luminescence of an ATP dilution series from 1.6 nM to 1.6 pM.

### Microbiological Carbon Production

Bacterial growth efficiency (BGE; synonym to carbon assimilation efficiency and carbon use efficiency) was estimated for the different treatment zones in the microcosms and for different time periods. BGE was calculated *via* the total net increase of TCC (taking into account also newly imported cells from feeding solutions and washed out suspended cells) vs. the total flux of DOC (import vs. export of DOC mass over time for the respective zones). Again, DOC flux and turnover (ΔDOC) were determined for the treatment zones in the microcosms (sediment layers receiving either DOM-1x or DOM-5x) where dilution by transverse mixing could be ignored (Figure 1).

### Bacterial Community Composition

Sediments for bacterial community analysis were collected when filling the microcosms (reference for t0), as well as at the end of the experiment (t171). At t171, sediment was collected from the individual segments of each treatment zone and pooled before DNA extraction and 454 deep amplicon sequencing. For extraction of total environmental DNA, we followed the extraction protocol of Pilloni et al. (2012). Purified DNA was stored at −20°C until further processing. For initial PCR reaction, the Primers Ba27f (5'-AGA GTT TGA TCM TGG CTC AG-3') and Ba907r (5'-CCT ATC CCC TGA GTT T-3') were used. For single direction reads, the 5'-end of the forward primer was fused with the specific sequence (5'-CGT ATC GCC TCC CTC GCG CCA TCA GXX XXX XXX XX-3'). For separation of different samples, the X-region was replaced by different multiplex identifier sequences (MID) recommended by Roche (Roche, Germany). Results from pyrosequencing were analyzed using Version 1.38.0 of the mothur software (Schloss et al., 2009). Since the data from each of the two replicate treatment zones were very similar, mean communities of DOM-1x and DOM-5x treated sediments are shown.

### Statistical Analyses

Statistical differences in community data were analyzed by a two-sided *t*-test with equal variance. Significance between different treatments along a timeline was analyzed using one-vector ANOVA with repeated measurements. Significance was assumed at *p* <0.05.

## Results

### Hydrological Flow Regime in the Microcosms

The conservative tracers amended to the two DOM feeding solutions, LiCl (DOM-1x) and KBr (DOM-5x), allowed to select zones for later sampling that were not or only marginally affected by transverse mixing (Figure 1). In consequence, water samples were collected only from the ports 2–4 (upper treatment zone) and 10–12 (lower treatment zone). Recovery of the conservative tracers in these zones was close to 100%. Similarly, sediment sampling during the experiment and at the end of the experiment focused only on these upper and lower treatment zones of the microcosms, respectively. Break-through curves of both conservative tracers revealed a mean water residence time of 14.8 h (data not shown).

### DOC and Nutrient Patterns

After the mixing of natural groundwater with fresh soil extract at the microcosms inlets, the two feeding solutions, i.e., DOM-1x with only a moderate amendment of DOC [+38% (phase 1) and +25% (phase 2)] and DOM-5x with a pronounced amendment [+440% (Ph1) and +715% (Ph2)], infiltrated the sediment microcosms. Feeding solution DOM-1x contained a DOC concentration of 1.90(±0.10) mg L^−1^ in experimental phase 1 and 1.57(±0.36) mg L^−1^ in phase 2. Feeding solution DOM-5x contained 5.15(±1.63) mg L^−1^ in Ph1 and 8.37(±0.54) mg L^−1^ in Ph2, respectively. The natural groundwater (GW) used in our study contained DOC at a concentration of 1.31(±0.42) mg L^−1^, exhibiting moderate fluctuations (min 1.09 mg L^−1^; max 2.03 mg L^−1^) throughout the 171 days of the experiment.

The concentration of dissolved nitrate (N-NO_3_^−^) and orthophosphate (P-PO_4_^3−^) in pristine groundwater ranged from 1.1 to 1.2 mg L^−1^ and from 23.3 to 59.9 μg L^−1^, respectively, over the course of the experiment. The DOM-1x and DOM-5x feeding solutions contained 1.1 mg L^−1^ (Ph1) and 1.2 mg L^−1^ (Ph2), as well as 1.3 mg L^−1^ (Ph1) and 1.9 mg L^−1^ (Ph2) N-NO_3_^−^, respectively. Values of P-PO_4_^3−^ ranged between 23.3 and 59.2 μg L^−1^ in DOM-1x, and 24.3 and 64.9 μg L^−1^ in feeding solution DOM-5x.

**Figure 2:**
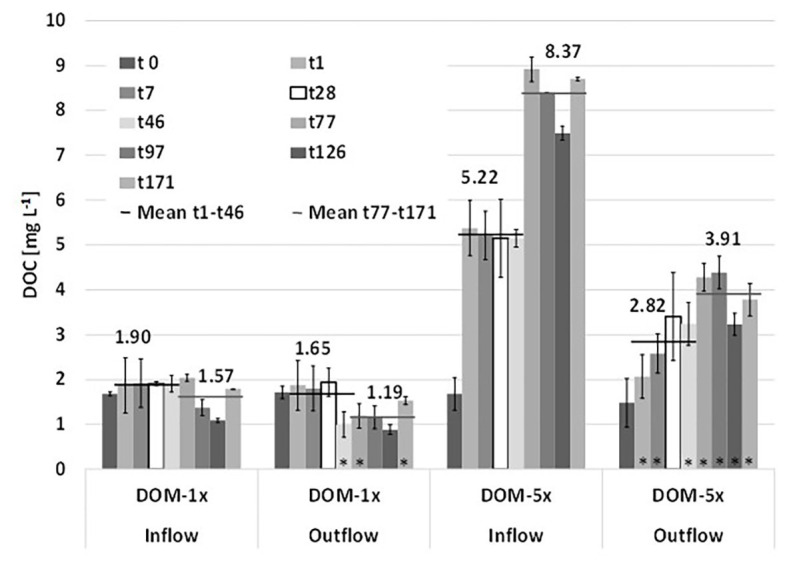
Depletion of dissolved organic carbon (DOC) during passage through the sediments of the two microcosms supplied with DOM-1x and DOM-5x. Data are mean values ± SD. Each of the two microcosm contained a DOM-1x and DOM-5x zone (biological replicates). For the individual sediment zones of each tank, additional technical replicate measurements were performed.

During passage through the sediments, DOM-1x was reduced by 23% (Ph1) and 24% (Ph2), respectively, in terms of DOC concentration. In the DOM-5x fed sediment zones, DOC was reduced by 35% (Ph1) and 53% (Ph2; Figure 2). In more detail, during early Ph1, the zones receiving DOM-1x exhibited no significant (*p* > 0.05) attenuation of DOC. Only later in Ph1 (>t28) and during the entire Ph2, the DOC concentration at the outlet of the DOM-1x fed sediments dropped below the inflow concentrations, showing a significant difference (*p* < 0.05) at t46, t77, and t171 (Figure 2). The sediments receiving DOM-1x did at no time provide evidence for quantitative sorption of DOC. In contrast, the sediments supplied with the DOM-5x solution, exhibited a significant (*p* < 0.05) reduction in DOC right from the beginning, however, with an outlet concentration that showed a quasi-exponential recovery within the first 4 weeks (t28). The difference between the outlet concentration at t1 and t28 is, besides a slowly establishing biodegradation activity, mainly referred to attenuation by abiotic sorption (Figure 2). For the following time period t28 until t171, adsorption ceased and the reduction of DOC was attributed to biological degradation.

Total DOC turned over (mineralization and assimilation) in the treatment zones accounted for 16.7 and 170.1 mg C in the DOM-1x and DOM-5x treatment zones, respectively, within the 171 days of the experiment. Calculations refer to an active sediment volume of 332.5 cm^3^ (= size of the individual treatment zones; see Figure 1), a porosity of 48%, a porewater volume of 159.6 ml and a mean water residence time of 14.8 h. In consequence, 1 L (dm^3^) of DOM-1x fed sediment mineralized on average 294(±230) μg C per day, the same volume of DOM-5x fed sediment mineralized 2.9(±1.1) mg C L^−1^_sed_ d^−1^. Values on DOC mineralization ignore the carbon assimilated into prokaryotic biomass, which was <5% in the DOM-1x treatment and 0.5% in the DOM-5x treatment (for more details see below).

### Dynamics in DOM Composition

**Figure 3:**
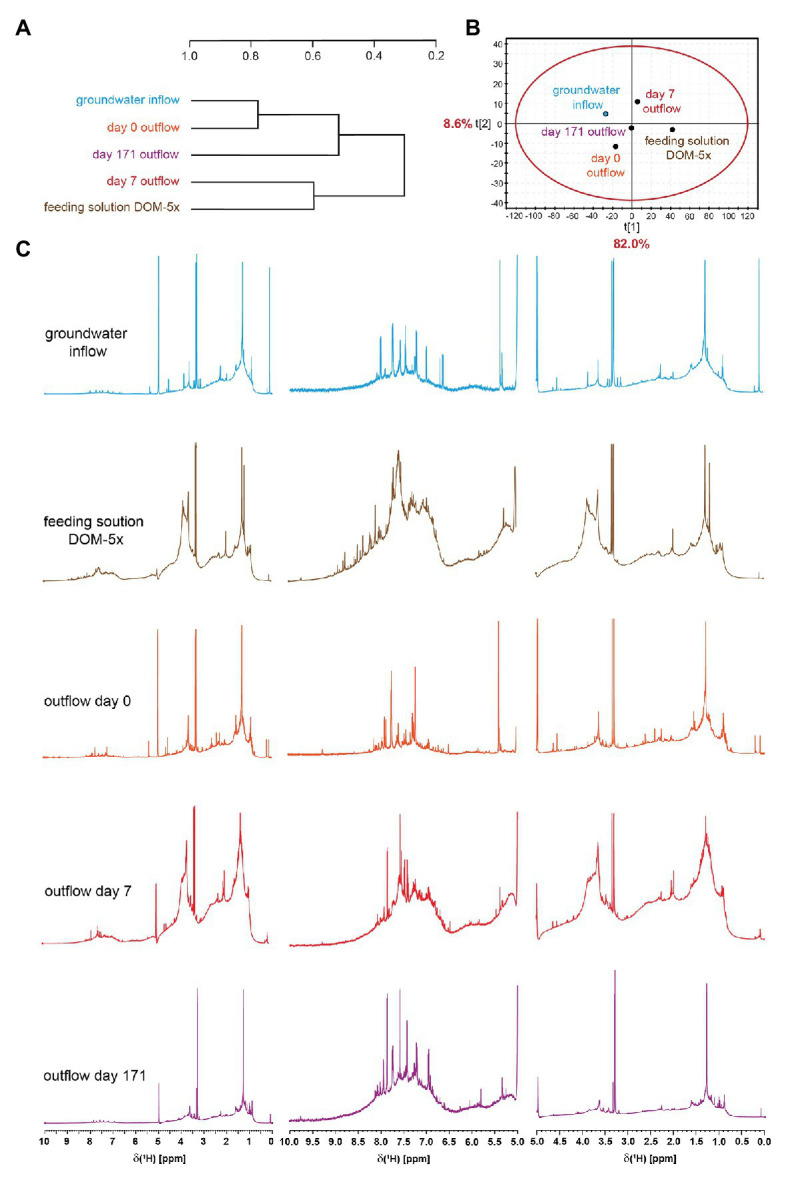
Hierarchical cluster analysis **(A)** and principal component analysis **(B)** derived from ^1^H NMR spectra (800 MHz, CD_3_OD) of porewater solid phase extracts (SPE)-DOM from DOM-5x **(C)** supplied sediments. HCA and PCA were computed with NMR section integrals of 0.01 ppm bucket width, with exclusion of HDO and HD_2_COD.

**Figure 4:**
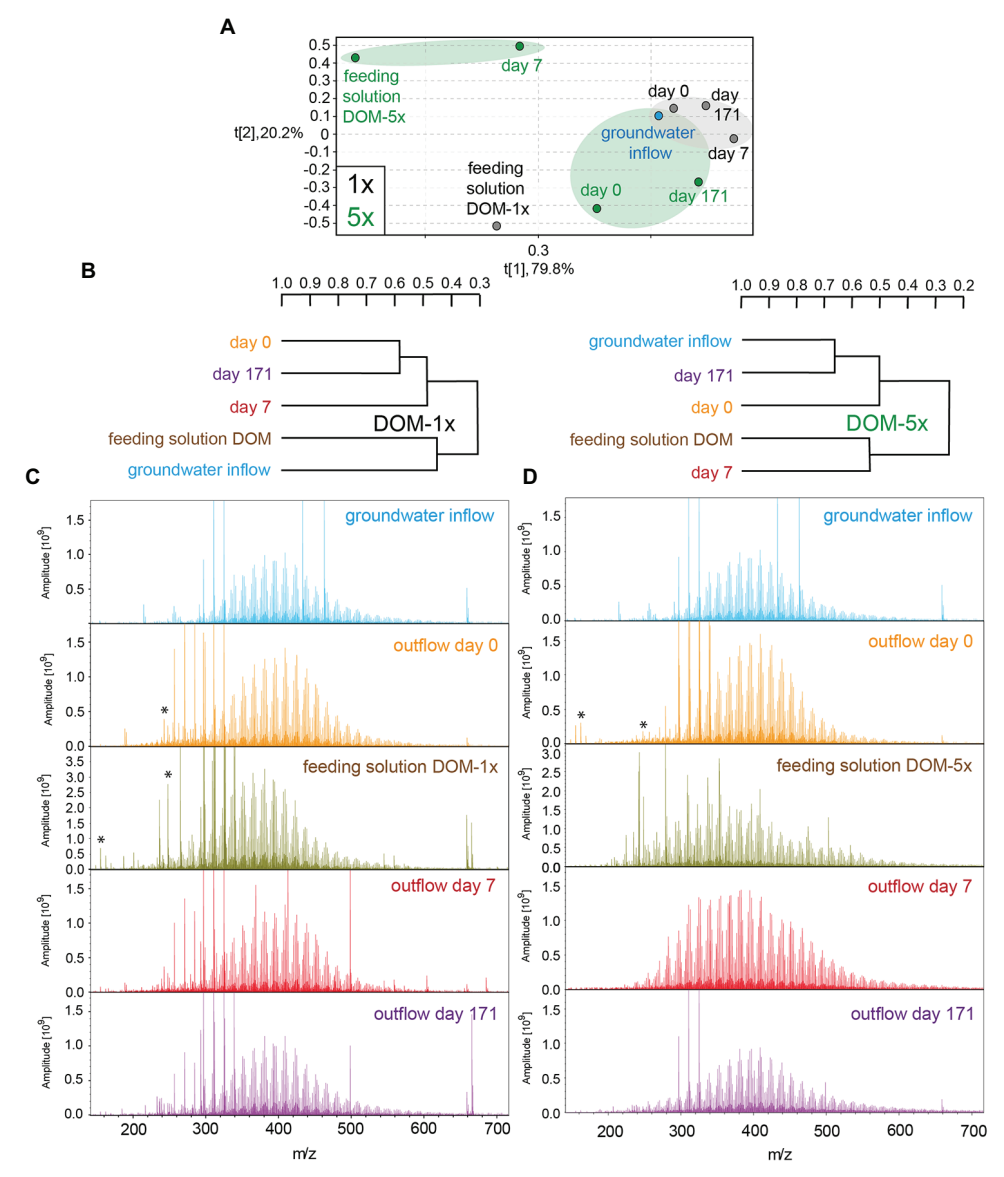
(−)ESI Fourier transform ion cyclotron resonance mass spectrometry (FT-ICR-MS)-derived principal component analysis **(A)** and hierarchical cluster analysis **(B)** of groundwater and DOM-1x and DOM-5x feeding solution and eluates at different time points. (−)ESI FT-ICR mass spectra of groundwater and DOM-1x (**C**; left panel) and DOM-5x (**D**; right panel) feeding solutions and eluates; asterisk: doubly charged ions.

High-field NMR spectroscopy and FT-ICR mass spectrometry demonstrated structural and compositional changes of DOM during passage through the microbial active aquifer sediments (Figures 3, 4). ^1^H NMR spectra, available for natural groundwater and the DOM-5x treatment, revealed a high similarity in DOM composition for the natural groundwater, and natural groundwater after sediment passage (t0), but clearly distinct chemical environments of the soil leachate DOM-5x as expected (Figure 3B). The eluate of DOM-5x fed sediments at t7 still exhibited an overall similarity to the feeding solution (Figure 3A) but showed a relative depletion of certain compound groups caused by sorption to sediment particles (Figure 3C and Supplementary Figure S1). After 171 days of operation (t171), the eluate from DOM-5x fed sediments collected at the microcosm outlets were most similar to natural groundwater (Figures 3A,B). Our results show that following a period of abiotic sorption and activation of the microbial communities, biodegradation processes in the DOM-5x fed sediments efficiently removed bioavailable DOM components originating from the soil leachate and turned the DOM from a soil leachate to a typical groundwater DOM in terms of relative abundance of key substructures. This finding was also strongly supported by the FT-ICR-MS analysis which revealed similar patterns for samples collected from the DOM-5x treatment zones (Figure 4C). In more detail, the NMR analysis showed that groundwater DOM featured the highest content of pure aliphatics (CCCH units; Table 1) and the lowest content of oxygenated aliphatic groups. In contrast, the soil leachate (feeding solution DOM-5x) was much more oxidized than the groundwater DOM and contained large fractions of potentially lignin-derived aromatic and phenolic units (Supplementary Figures S2, S3). The group of oxygenated aliphatics included a large complement of carbohydrates, aromatic and aliphatic methyl esters and methyl ethers, peptides, and carboxyl-rich alicyclic matter (Supplementary Figure S3). After 1 week of operation of the microcosms, DOM-5x that has passed the active sediments showed a selective depletion in aromatic methyl esters and -ethers by ~35% (Supplementary Figure S1C), in line with a known preferential sorption of aromatic compounds (Chefetz and Xing, 2009; Young et al., 2018; Subdiaga et al., 2020). After 171 days of experiment, the long-term supply of DOM-5x has led to an altered dynamic equilibrium, with increased proportions of aromatic and olefinic molecules as well as aliphatic methyl esters, compared with the DOM from t0 (Supplementary Figures S1A,B). In fact, these compounds, initially attenuated by sorption, now quantitatively passed the sediments. A more detailed description of spatiotemporal dynamics in ^1^H NMR spectra is provided in the SI.

**Table 1:** Section integrals for key substructures [derived from ^1^H NMR spectra (800 MHz, CD_3_OD) of microcosm SPE-DOM (PPL; see also Supplementary Figures S2, S3)].

δ(^1^H)	Key substructures	Natural groundwater inflow (t0)	Natural groundwater outflow (t0)	Feeding solution DOM-5x inflow (t0–t171)	Feeding solution DOM-5x outflow (t7)	Feeding solution DOM-5x outflow (t171)
10–7 ppm	C_sp2_H	3.0	3.6	8.2	4.4	4.5
7–5 ppm	phenols, O_2_CH	0.8	1.4	5.4	4.0	2.8
5–3.1 ppm	OCH	13.9	17.2	36.3	28.1	21.1
3.1–1.9 ppm	OCCH	28.0	25.4	20.4	25.1	23.9
1.9–0.5 ppm	CCCH	54.3	52.4	29.7	38.4	47.8

Key substructures represent aromatics and olefins with polarized double bonds (*δ_H_* ~10–7 ppm); phenols and olefins and anomeric positions in carbohydrates (*δ_H_* ~7–5 ppm); oxygenated aliphatic groups, with oxygen-containing functional groups (*δ_H_* ~5–3.1 ppm); amines, aliphatic groups, with oxygen-containing functional groups 3 bonds away, e.g., carboxylic acids (*δ_H_* ~3.1–1.9 ppm), “purely” aliphatic groups, with oxygen >4 bonds away (*δ_H_* ~1.9–0.5 ppm).

FT-ICR mass spectra of feeding solution DOM-5x showed a higher mass range and a more complex pattern than all other samples (Figure 4C), in line with the prominent signature of oxygenated aliphatic and aromatic functional groups observed with the NMR spectra (Figure 3C and Supplementary Figure S1). FT-ICR-MS revealed a different temporal evolution of DOM processing for CHO, CHNO, CHOS, and CHNOS molecular series in DOM-1x and DOM-5x treatments (Figure 4A and Supplementary Figures S4, S5).

The temporal evolution of the average mass in DOM-5x treatments was consistent with a continual degradation from larger to smaller molecules (Supplementary Table S2). For example, turnover of CHO compounds between t0 and t7 shifted toward smaller molecules and slightly more deoxygenation at otherwise similar H/C ratios (H/C ratio ~0.8–1.5). At t171, the group of oxygenated, lignin-like compounds of relatively high mass (m/z > 450 Da) was depleted (Supplementary Figures S4, S5). Furthermore, only highly oxygenated CHO (with m/z < 450) and CHNO compounds (O/C ratio > 0.4) disappeared. Selectively processed CHOS molecules were lipid-like and of weak to considerable unsaturation. More details on FT-ICR-MS results are provided in the SI.

The FT-ICR-MS analysis of the DOM-1x treatment demonstrated that the minor amendment of soil extract to natural groundwater (feeding solution DOM-1x) did not cause a significant change in overall DOM composition (Figure 4C). Apart from that, the DOM in the water samples collected after sediment passage at t0, t7, and t171 was very similar to that in natural groundwater and the feeding solution DOM-1x, as revealed from a hierarchical cluster analysis and a principal component analysis (Figures 4A,B). No evidence was found for quantitative sorption of DOM in the early phase of the experiment with samples from the DOM-1x treatment. However, minor compositional changes pointed at an attenuation of some large and highly oxygenated molecules (Supplementary Figure S4). With an ongoing adaptation of the sediment microbial communities, molecules with a high mass, high oxygenation, and unsaturation were selectively depleted. Lignin-like CHO and CHNO compounds and lipid-like CHOS compounds were reduced in relative intensity (Supplementary Figure S4).

### Changes in Bacterial Biomass and Activity

**Figure 5:**
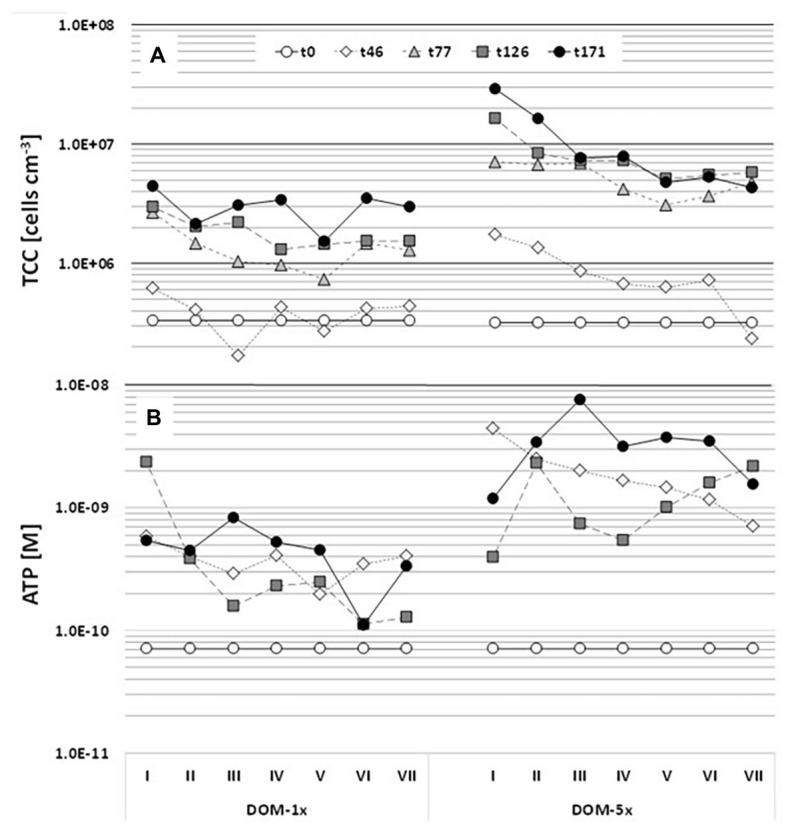
Dynamics of sediment bacterial biomass **(A)** and activity **(B)** over 171 days of incubation. The microcosms were infiltrated by the DOM-1x and DOM-5x feeding solution from left to right, entering the microcosms at sector I and leaving at sector VII. Samples were collected from the top sediment layer of each microcosm. Values are means of duplicate measurements of technical replicates.

The natural groundwater that constituted the major part of the feeding solutions contained 7.4 × 10^4^(±6.4 × 10^4^) cells ml^−1^. The abundance of bacterial cells attached to the aquifer sediment at the beginning of the experiment was 3.7 × 10^5^(±5.7 × 10^4^) cells cm^−3^ sediment. Assuming a sediment porosity of 48%, this translates into a ratio of attached to suspended cells per unit sediment volume of 11:1. In the regularly sampled DOM-1x receiving sediments, the number of attached cells remained close to the starting conditions in the early phase (Ph1) of the experiment (≤46 days) with only a slight in‐ or decrease in the individual segments by 2 to 4-fold (Figure 5A). Only from t77 on until t171, the sediment TCC substantially increased, to a final 13-fold higher cell concentration (4.42 × 10^6^ ± 3.47 × 10^5^ cells cm^−3^) at the inlet (segment I) and a 9-fold higher number (2.96 × 10^6^ ± 1.3 × 10^5^ cells cm^−3^) at the outlet (segment VII; Figure 5A). Sediment cell numbers determined at t77, t126, and t171, although still slightly rising with time did not reveal a statistically relevant difference (*p* < 0.05). Similarly, at the end of the experiment (t171), sediment, cell numbers at the inlet and outlet of the DOM-1x zones showed no significant difference (*p* > 0.05). A linear decrease of cell numbers from the sediment at the inlet to the sediment at the outlet statistically best described the final pattern (Supplementary Figure S6). In fact, the period of most pronounced growth of sediment bacteria was between t46 and t77 (Figure 5A).

Sediment bacterial biomass developed rather differently in the DOM-5x receiving sediment zones. Already during the early phase (Ph1) of the experiment (≤46 days), the sediment TCC exhibited a pronounced increase that continued until t171 with a maximum concentration of 2.9 × 10^7^(±6.45 × 10^5^) cells cm^−3^ (92-fold increase) at the microcosm inlet. In segment VII at the outlet, sediment TCC reached 4.32 × 10^6^(±1.9 × 10^5^) cm^−3^ and thus a 14-fold increase in comparison to the starting concentration. Even more pronounced than with the DOM-1x treatment, the sediment bacterial biomass started to level off with t77 (Figure 5A). The period of most significant growth of sediment bacteria was between t1 and t77. Opposite to the DOM-1x sediment, TCC were found significantly different (*p* < 0.05) between the segments close to the inlet and outlet at the end of the experiment. An exponential decrease in cell numbers from segment I to VII statistically best described the pattern observed at t171 (Supplementary Figure S6).

The activity of the sediment bacterial community, measured by total ATP, increased by a factor of 10 in the DOM-1x supplied zones already in the early phase of the experiment (≤46 days), even before an increase in biomass (TCC) was observed (Figure 5B). Later, ATP values showed no further remarkable changes until the end of the experiment (t171), irrespective of the increase of biomass. The spatial distribution of microbial activity exhibited a trend of decreasing activity from the inlet to the outlet of the microcosm (inlet value about 40% higher at t171), however, values in inlet segment I and outlet segment VII were not significantly different (*p* > 0.05). Microbial activity patterns developed differently in the sediments receiving DOM-5x. First, activity rose more than 60-fold in the inlet segment within the first 46 days of the experiment compared with a 10-fold increase in the outlet segment. This time, however taking the coarse temporal resolution into account, the rise in activity was parallel to an increase in biomass (Figure 5), at least in the sediments close to the microcosm’s inlet. At t171, the spatial distribution of bacterial activity across the microcosm exhibited similar values in the inlet and outlet sediments.

The direct comparison of the sediment bacterial abundance after 171 days for the two treatments, i.e., supply with DOM-5x and DOM-1x feeding solution, revealed 7-fold higher cell counts in the DOM-5x sediments close to the inlet and 1.5-fold higher counts at the outlet (Supplementary Figure S7). The comparison of sediment bacterial activities showed 5–30-fold higher values in the segments I and II, and 2-fold higher activities close to the outlet for DOM-5x treatment (Supplementary Figure S7).

**Figure 6:**
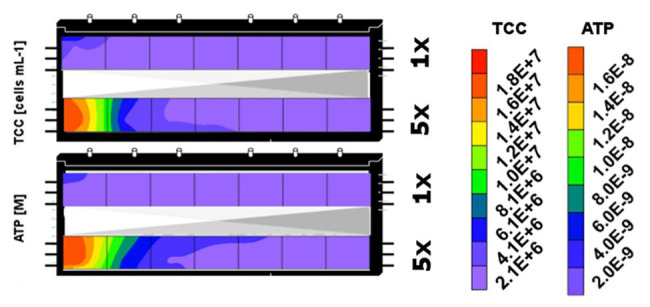
Spatial distribution of sediment bacterial biomass (TCC) and activity (ATP) in the sediment zones that received DOM-1x (here upper segment) and DOM-5x (lower segment) feeding solution at the end of the experiment (t171). The contour plots combine data from sediment segments of the two microcosms that were subject to similar DOM supply.

For the 171 days of experiment, samples could be collected only from the top sediments of the two microcosms, resulting in only a single set of samples from the individual segments (I–VII) and treatments (DOM-1x and DOM-5x). However, at the end of the experiment (t171), the two microcosms were sacrificed to provide biological duplicate measurements of the two treatments (Figure 6). In fact, the bacterial biomass in the sediments that have received DOM-1x coincided well between microcosm 1 and 2; no significant differences were calculated (*p* > 0.05). A similar result was obtained for the DOM-5x receiving sediment segments, however, here with moderately higher TCC in microcosm 2; again not significantly different (*p* > 0.05) from microcosm 1. Figure 6 shows the final situation for sediment bacterial abundance (TCC) depicted as mean values from two replicate zones receiving either DOM-1x or DOM-5x feeding solution.

If one wants to estimate the total number of bacterial cells, and thus bacterial carbon produced during the course of the entire experiment, attached and suspended cells need to be considered in combination. The number of cells introduced to the microcosms already mentioned above, was in the range of 7.4 × 10^4^(±6.4 × 10^4^) ml^−1^. The number of bacterial cells continuously washed out from the microcosms was very stable over the entire experiment and, surprisingly, in the same range for the sediment zones receiving DOM-1x and DOM-5x, accounting for 9.30 × 10^5^(±5.20 × 10^5^) and 9.03 × 10^5^(±5.04 × 10^5^) ml^−1^, respectively (Supplementary Figure S8). In comparison to the cells continuously imported into the microcosms, the number of exported cells were significantly higher (*p* < 0.05) by more than one order of magnitude. Since the total amount of cells transported out of the microcosms exceeded the increase of biomass in the sediments by far, the total bacterial biomass produced in the sediments receiving DOM-5x was only marginally higher than in sediments receiving DOM-1x (see below). In consequence, BGE was higher in DOM-1x fed bacterial communities.

### Bacterial Carbon Production and Bacterial Growth Efficiency

Bacterial carbon production was estimated from the overall increase of bacterial cells (ΔTCC) taking into account the import and export of suspended cells, as well as the dynamic change in sediment associated bacteria. Data refer to a specific sediment volume of 332.5 cm^3^ (= treatment zone; see Figure 1) with a porewater content of 159.6 ml assuming 48% porosity, and a mean water residence time of 14.8 h. Over the entire experiment, the DOM-1x fed sediment segment produced 3.89 × 10^10^ cells equal to 780 μg C, applying a conversion factor of 20 fg C cell^−1^. The DOM-5x fed sediment segment produced 4.06 × 10^10^ cells equal to 810 μg C. If one analyzes the overall carbon production for the individual phases of the experiment, it becomes clear that the significant differences with DOM-1x and DOM-5x fed sediments found in the number of attached cells (Figures 5A, 6 and Supplementary Figure S7A) are of only minor importance for overall production. The DOM-1x fed sediments produced on average 13.7(±4.8) μg biomass C L^−1^_sed_ d^−1^. The DOM-5x fed sediments produced 14.3(±3.5) μg biomass C L^−1^_sed_ d^−1^. Taking into consideration the quite similar BCP and the different amounts of organic carbon mineralized in the DOM-1x and DOM-5x treatments, striking differences are observed for the efficiency bacteria assimilated the organic carbon.

The bacterial carbon use efficiency (CUE) and thus the BGE were higher with the bacterial communities in the sediments receiving less in total and less in fresh DOM. When calculated from the dynamic changes in DOC and TCC, the BGE for the entire experiment with the DOM-1x treatment was 4.7% (Table 2). Evaluating the different phases of the experiment, the sediment bacterial communities performed quite efficient at the beginning, i.e., BGEs of 8.9% between t0 and t46 and 10.4% for t28–t46. Lower values were calculated for the early second phase (Ph2). Toward the end of the experiment, a value of 8.9% (t126–t171) was obtained (Table 2).

**Table 2:** Bacterial growth efficiencies (BGE) in the sediments fed by DOM-1x and DOM-5x, respectively, and individual periods of incubation.

Experimental phase	BGE (%)			
	1x		5x	
	Sediment at microcosm inlet	Sediment at microcosm outlet	Sediment at microcosm inlet	Sediment at microcosm outlet
Experiment (t_0_–t_171_)	4.7		0.5	
Phase 1 (t_0_–t_46_)	8.9		0.8 (1.1)^*^	
Phase 1B (t_28_–t_46_)	10.4		2.5 (2.6)^*^	
Phase 2A (t_46_–t_77_)	1.2		0.4	
Phase 2B (t_77_–t_126_)	3.0		0.2	
Phase 2C (t_126_–t_171_)	8.9		0.4	

^*^BGE values were calculated from BCP (∆TCC) and DOC mineralization (∆DOC).

The BGEs for the communities calculated from ∆DOC/∆TCC in the DOM-5x fed sediments revealed a mean value of only 0.5% for the entire experiment. Here, at the beginning, i.e., t0–t46 and t28–t46, the BGE was around 1 and 2.5%, respectively. In the second phase of the experiment with the higher DOM import, the BGE dropped to 0.2–0.4% (Table 2).

### Carbon Utilization Patterns

**Figure 7:**
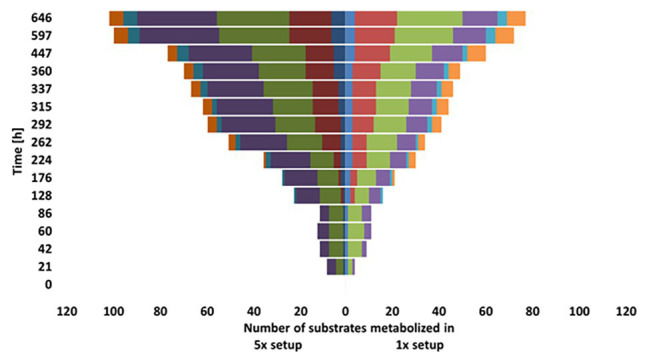
Comparison of substrate utilization by the microbial communities from the DOM-1x and DOM-5x fed sediments.

Our tests for the metabolic flexibility in using different carbon sources of the groundwater microbial communities washed out of the DOM-1x and DOM-5x fed sediments at t171 revealed several differences. In the early phase of incubation of the carbon utilization test system (≤86 h), signals of pronounced degradation were obtained only for carbohydrates and carboxylic acids, with the DOM-5x fed communities being metabolically more versatile in the beginning (Figure 7). With a delay (≥128 h), transformation of amines increased in importance and degradation of amino acids started. Here, initially (until 224 h) the DOM-1x communities utilized a greater substrate diversity. The conversion of polymers started first (176 h) with the microbial communities originating from the DOM-1x fed sediments and remained most pronounced throughout the entire incubation (Figure 7). The differences in usage of different groups of substrates within the two communities were statistical significant for the respective substrate groups (ANOVA, *p* < 0.05).

### Dynamics in Microbial Community Patterns

**Figure 8:**
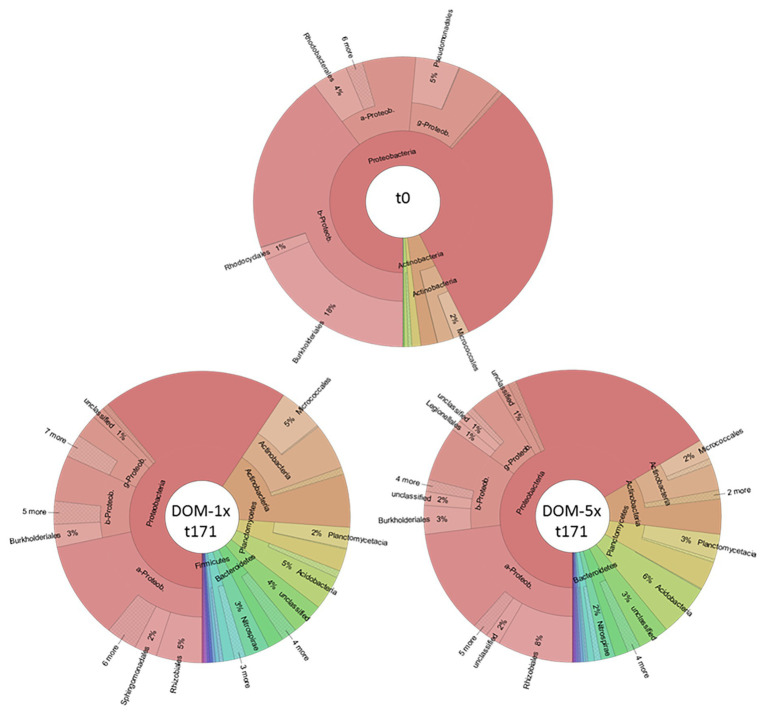
Bacterial community composition in fresh aquifer sediment and in the sediments receiving DOM-1x and DOM-5x for 171 days.

At the end of the experiment (t171), the individual segments of the treatment zones were sampled for sediments and analyzed together with samples from before the DOM treatment (t0) by 454 deep amplicon sequencing. At the start of the experiments, the sediment bacterial community was dominated by Proteobacteria (93%). Within this phyla, *Betaproteobacteria* were most prominent (43%), followed by *Alphaproteobacteria* (12%) and *Gammaproteobacteria* (10%). Other identified phyla included *Actinobacteria* (5%) and *Bacteroidetes* (1%; Figure 8).

In the bacterial communities of the DOM-1x sediments from day 171, the fraction of Proteobacteria accounted for 59%, exhibiting a significant decrease in proportion compared to t0 (*p* < 0.05). Moreover, the former dominating *Betaproteobacteria* (now 17%) were taken over by the *Alphaproteobacteria* (37%). The fraction of *Gammaproteobacteria* remained rather stable (9%). *Actinobacteria* (now 17%) as well as *Bacteriodetes* (now 3%) gained importance. The decline in Proteobacteria was accompanied by the emergence (>1% relative abundance) of other phyla such as *Planctomycetes* (5%), *Acidobacteria* (5%), *Nitrospirae* (3%), and *Firmicutes* (2%; Figure 8).

A similar change in dominance within the Proteobacteria was also observed for the DOM-5x sediment communities. Here, Proteobacteria declined to a relative proportion of 66% with *Alphaproteobacteria* (35%) being most abundant followed by *Betaproteobacteria* (18%), *Gammproteobacteria* (10%), and *Deltaproteobacteria* (2%). Also the patterns of the other phyla were very similar to the DOM-1x sediment community. In detail, *Actinobacteria* accounted for 11%, *Planctomycetes* for 6%, *Acidobacteria* for 6%, *Bacteroidetes* for 3%, and *Nitrospirae* for 2% of relative abundance (Figure 8). Jaccard’s similarity of the two treatments, DOM-1x and DOM-5x, was 0.882.

Based on the deep-sequencing data, we also determined the bacterial richness, the Shannon-Wiener diversity, and the evenness. After 171 days, the bacterial richness and diversity was found considerably increased in the inlet area of the sediments fed with the DOM-1x and DOM-5x solutions in comparison to the untreated fresh sediment at t0 (Supplementary Figure S9). The DOM-5x sediments exhibited highest values with an *R* = 139 and a *H*' = 3.8, while the DOM-1x sediments revealed an *R* = 92 and *H*' = 3.5. With distance to the inlet, these measures declined in both treatments (DOM-1x and DOM-5x). However, while in the DOM-1x treated sediments values at the microcosms outlet area did not significantly differ (*p* > 0.05) from the starting conditions (*R* = 32, *H*' = 2.7), the bacterial communities in the DOM-5x sediments revealed an elevated diversity throughout the microcosms (Supplementary Figure S9B).

## Discussion

Apart from the comparable low concentrations of microbial cells per volume, groundwater ecosystems constitute a significant reservoir of organic carbon and biomass due to its enormous dimensions, which by far exceed those in soils and surface waters (Magnabosco et al., 2018). However, because of its difficult accessibility, organic matter transformation and its linkage to prokaryotic production in oligotrophic aquifers have rarely been investigated in detail. Our study in sediment flow-through model aquifers is a first step toward a better understanding of ecosystem functioning with emphases on carbon turnover and microbial carbon production, physiological adaptations, and biodiversity. This study revealed several important findings. First, DOM from soil extract, simulating a pulse of organic matter entering the groundwater system at times of heavy rain and high groundwater recharge, was, after an initial phase of abiotic sorption, substantially degraded along with an increase in sediment associated bacterial biomass and activity. In contrast to sediments that received DOM characteristic for oligotrophic groundwater in terms of concentration and composition, the fresh soil OM in DOM-5x supplied sediments led to a much higher standing stock of prokaryotic biomass. However, the overall biomass production, taking into account the biomass that was continuously transported out of the microcosms by water flow, was only slightly different between the two treatments, pointing at a much lower carbon use efficiency of the microbial communities supplied with the soil extract that contained a more easily degradable share. Second, passage through 1 m of conditioned microbial active aquifer sand turned the DOM from soil leachate to typical groundwater DOM in terms of composition and structural features. Furthermore, onset of pronounced conversion of DOM as well as bacterial growth took a few weeks rather than hours or days, with the early period of the experiment characterized by DOM sorption and an increase in cell-specific microbial activity uncoupled from growth. Finally, a higher diversity in DOM composition fostered a higher bacterial diversity. With respect to physiological adaptation, microbial communities that have seen only the more stable and refractory DOM from oligotrophic groundwater performed better in degrading more complex organic compounds. The individual findings are briefly discussed in the following in comparison to the state of knowledge.

### DOC Removal and Microbial Growth With Variable DOM Supply

Soil and sediment layers covering groundwater ecosystems protect the subterranean environment from surface related impacts. At the same time, they shield the down below habitats and its communities from important energy sources, i.e., organic carbon, originating from surface terrestrial and aquatic environments (Griebler et al., 2014; Hofmann and Griebler, 2018). During its passage to the aquifer, organic carbon in soil seepage water and surface water recharging aquifers is significantly reduced in concentration and easy degradable compounds (Fredrickson et al., 1989; Kalbitz et al., 2000; Pabich et al., 2001; Shen et al., 2015). In consequence, oxic aquifers are typically oligotrophic (of low productivity) and hold groundwater that is poor in organic carbon (Regan et al., 2017; Hofmann and Griebler, 2018). These conditions are also mirrored in the comparably low abundance and activity of prokaryotic cells, 10–100-times less than in oligotrophic surface waters (Pedersen, 2000; Pedersen et al., 2008; Griebler and Lueders, 2009; Fillinger et al., 2019).

Currently, only a few studies have addressed the turnover of organic carbon in non-contaminated, oligotrophic aquifers in terms of concentration (Baker et al., 2000; Hofmann and Griebler, 2018) and composition (Leenheer, 2002, Einsiedl et al., 2007; Longnecker and Kujawinski, 2011; Shabarova et al., 2014; Pracht et al., 2018), as well as the linkage of DOM mass and quality to microbial growth and secondary production (Hofmann and Griebler, 2018; Wu et al., 2018). Moreover, most studies were carried out in batch or only considered the mobile groundwater phase, neglecting the sediment matrix as important place for microbial life and sorption for organic matter.

Our study addressed the fate of DOM from soil seepage water and groundwater when passing through about 1 m of microbial active sandy aquifer sediment. Additionally, the prokaryotic growth and productivity was evaluated over time in the water and sediment matrix. For the reason of comparison, we simulated two scenarios. First, an oligotrophic aquifer that is infiltrated with natural groundwater which is only very moderately amended with DOM originating from soil; a situation that mimics a period of low groundwater recharge (termed DOM-1x). Second, we simulated the import of DOM from soil at an elevated concentration and different in composition; a situation that mimics a long-term recharge event initiated by periods of heavy rain (termed DOM-5x).

Similar to earlier observations in batch experiments with DOC-poor groundwater (Hofmann and Griebler, 2018), it took several weeks (>28 days) until a significant reduction in DOC concentration was observed in the sediments supplied with DOM-1x (Figure 2). The subsequent DOC removal of 23–24% is slightly less compared to the original 25–38% amendment of soil extract to groundwater in DOM-1x. The aquifer sediments that received mainly soil extract with an elevated DOC concentration (DOM-5x) exhibited a faster onset of bacterial growth and increase in sediment prokaryotic biomass (Figure 5A). The pronounced attenuation of DOM observed right from the beginning is attributed to adsorption of selected DOM fractions (Figure 2). Later, ≥t28, sorption processes lost importance and DOC removal was driven mainly by microbial mineralization. No evidence was gained for quantitative sorption in the DOM-1x fed sediments (Figure 2) which showed an average DOC removal rate of 294(±230) μg C L^−1^_sed_ d^−1^, 10-times lower than in the DMO-5x sediments [2.9(±1.1) mg C L^−1^_sed_ d^−1^].

Our findings are in agreement with own earlier observations from groundwater batch experiments. In detail, the DOC-poor groundwater in oligotrophic aquifers sustained a comparably low growth and productivity (Hofmann and Griebler, 2018). DOM when mobilized from soil and entering the aquifer come along with some labile fractions (Pracht et al., 2018). However, aquifer sediment microbial communities that see DOM pulses for only short (hours to days) and irregular intervals with interruptions longer than the pulses, are hypothesized not able to spontaneously respond. Dependent to the DOM quality, it may take several days to a few weeks until pronounced carbon turnover establishes. Even more delayed is the growth of microbes and buildup of a higher biomass standing stock (Hofmann and Griebler, 2018). Similar findings come not only from groundwater batch experiments but also from managed aquifer recharge sites (Foulquier et al., 2010, 2011). These latter studies emphasize that the typical short exposure time of microorganisms to DOM pulses from land surface and soil may not allow substantial microbial growth and carbon removal. The observation that higher loads of DOM from the soil and vadose zone rapidly disappear with distance and depth in the aquifer led us to the conclusion that dissolution (Foulquier et al., 2010) and sorption (Leenheer, 2002; Pracht et al., 2018) are most responsible for the fast attenuation of DOM in the initial phase of pulses.

In transition zones that receive a continuous flux of bioavailable DOM, the preparedness of microbial communities in terms of active biomass and biodegradation plays an important role. Our results invite speculations that these zones are characterized by a higher standing stock of microbial biomass but are rather small in spatial dimension. In the microcosms used in our study, most of the prokaryotic biomass newly formed was, after 171 days, concentrated in the first 25 cm of sediment from the inlet (segments I and II; Figures 5A, 6). While in the DOM-5x fed sediments, an exponential decrease of microbial biomass with distance to the microcosm inlet was observed (Supplementary Figure S6), there was no clear pattern in sediment prokaryotic cell numbers of zones receiving DOM-1x. The slight decrease in cell abundance with distance to the inlet could be described by a linear function (Supplementary Figure S6).

It should also be mentioned that the batch study of Wu et al. (2018) drew a different picture of a groundwater microbial community in which, with a delay of only 1.5 days, microbes responded to sediment derived DOM supply in terms of organic matter removal and growth. Moreover, the bacterial density increased extremely fast by two orders of magnitude (from 10^5^ to 10^7^ cells ml^−1^) in the first 8 days along with a decline of DOC from about 8 to 4 mg L^−1^. While the DOC concentration then stayed constant for the rest of the experiment (50 days in total), cell counts, after peaking at day 8, dropped again 3-fold until day 13 before leveling off (Wu et al., 2018).

Evident from the sediment bacterial abundance data, pronounced bacterial growth in the DOM-1x fed sediments took place only following phase 1 between t46 and t77. In the DOM-5x fed sediments, bacterial growth started already earlier, i.e., in experimental phase 1. Later (>t77), growth slowed down, indicating the achievement of a treatment-specific microbial carrying capacity. At constant sediment bacterial biomass, growth is reflected in the net cell numbers (suspended cells in the outlet minus suspended cells in the inlet) that are continuously washed out. Data obtained point at rather similar cell production rates in both treatments, DOM-1x and DOM-5x, toward the end of the experiment. In conclusion, the differently supplied sediment communities overall produced a similar biomass with microbes in DOM-5x sediments assimilating 10-times less carbon compared with those in DOM-1x sediments.

Growth and translocation of bacterial populations in both treatments revealed interesting spatio-temporal dynamics. While a pronounced share in cell numbers was in the early phases mainly concentrated to the inlet area of the microcosms, zones exhibiting increasing cell numbers later shifted toward the outlet, as observed for the DOM-5x sediments. It is very likely that the increase of prokaryotic cells more distant to the inlet was mainly caused by cells that were produced in the inlet area but after release to the pore water settled down further downstream (Grösbacher et al., 2018).

There are a number of groundwater studies that report a positive correlation between the number of suspended bacterial cells and DOC, but sediment studies are rare. Li et al. (2012, 2013) obtained a positive correlation between microbial density and DOC as well as bioavailable DOC (BDOC) concentration. In their experiments, Li et al. (2013) observed a significant decline in BDOC and biomass along the water flow path. The potential of soil DOM as organic carbon source that carries a significant fraction of BDOC and its stimulatory effects to bacterial production and DOC degradation were underlined in mesocosm studies by Judd et al. (2006). Here, soil water DOM enhanced lake and stream bacterial production by 320–670% relative to controls (Judd et al., 2006).

### Composition of DOM and Changes With Sediment Passage

NMR and FT-ICR-MS analyses confirmed that soil leachate, not surprisingly, was very different in DOM composition and molecular structures when compared to groundwater. The soil leachate contained a prominent fraction of ready bioavailable organic compounds which, following an acclimatization period of several weeks, was quantitatively transformed and mineralized by the aquifer sediment microbial communities. DOM from soil leachate, after passage of 1 m of active aquifer sediment, resembled the DOM in natural oligotrophic groundwater in terms of composition and key substructures. In general, with time and distance to the source, a continual shift from larger to smaller molecules and a defunctionalization of the soil leachate DOM took place. Oxygen-containing functional groups in aromatic (*δ_H_* ~6.5–7 ppm) and aliphatic (*δ_H_* ~3.7–4.5 ppm) molecules, including carbohydrates and peptides were preferentially degraded (Figure 3C). Oxygenated lignin-like and tannine-like compounds (*δ_H_* > 6.5 ppm) were attenuated and degraded faster in time than lipid-like and less oxygenated compounds which exhibited a selective depletion only in the late phase of the experiment (t171; Supplementary Figures S4, S5). A set of highly oxygenated aliphatics, most probably substituted carbohydrates, resembled microbial metabolites and underlined bio-processing of DOM. Worth mentioning, in the early phase of soil leachate DOM passing through the aquifer sediment, biodegradation activity was still minor and superimposed by adsorption to the sediment matrix, indicated by a depletion of all types of oxygenated aromatic compounds at t7. Later, at t171, a distinct processing of unsaturated molecules was observed: ^1^H NMR spectra indicated the selective enrichment of aromatic compounds, suggesting a selective preservation in comparison with the aliphatic compounds. Attenuated in the early phase of the experiment by their higher propensity for adsorption, they started to re-appear with a fading out of sorption processes. The groundwater DOM at a typically low concentration, on the other hand, as inferred from DOM concentration and composition data, was not subject to pronounced sorption nor biodegradation in the early phase of the experiment. It generally contained higher proportions of aliphatic molecules than the soil leachate. Moreover, a higher abundance of carboxyl-rich alicyclic molecules (Supplementary Figure S1D) pointed at an overall higher extent of carboxylation, and depletion of other highly oxygenated molecules (Hertkorn et al., 2006). A similar pattern has been observed in an earlier study on DOM transformation in a karst groundwater ecosystem (Einsiedl et al., 2007).

Recent publications have described complex relationships between community composition and temporal evolution of DOM during microbial processing (Shabarova et al., 2014; Logue et al., 2016; Wu et al., 2018). These are reflected in our findings as well and show a complex multistage evolution of DOM under concomitant variation of microbial abundance and diversity (see below).

### Microbial Activity, Bacterial Production, and Carbon Use Efficiency

The activity and productivity of microbes in energy poor, oligotrophic groundwater environments is considered extremely low (Thorn and Ventullo, 1988; Albrechtsen and Winding, 1992; Kazumi and Capone, 1994; Phelps et al., 1994; Wilhartitz et al., 2009). Evidence from several studies indicates that respiration (catabolism) and biomass production (anabolism) often are uncoupled in oligotrophic ecosystems (Teixeira de Mattos and Neijssel, 1997; Carlson et al., 2007; del Giorgio and Newell, 2012). Main reason may be a particular limitation (e.g., organic carbon and nutrients) that prevents cells from biomass production and multiplying. In batch experiments with groundwater microbial communities, Hofmann and Griebler (2018) observed a comparable high ATP level in cells that were respiring DOC but were actually non-growing because of P limitation. Similarly, our current experiment showed that attached prokaryotic cells first increased their cell-specific activity, as interpreted from an early rise in ATP concentration (Figure 5B), before pronounced growth took place with a considerable delay. Further evidence for a pre-phase of activation ahead of reproduction is the fact that DOC reduction started in advance to an increase in cell numbers. Even later, when growth started, only a small fraction of the carbon consumed was converted into new biomass, expressed as BGE or CUE (del Giorgio and Newell, 2012). While values of BGE in different aquatic environments typically range from 5 to 50% (del Giorgio and Cole, 1998; Manzoni et al., 2012), eventually lower BGE values have been reported for extremely energy poor environments and/or microbial communities limited in nutrients (Reinthaler et al., 2006; del Giorgio and Newell, 2012), including oligotrophic groundwater (Hofmann and Griebler, 2018). Our current study underlines that the availability of labile DOM indeed leads to a pronounced DOC mineralization; but with a delay of days to weeks, and not necessarily coupled to significant growth. Indeed, the sediments receiving increased concentrations of soil leachate (DOM-5x) established a much higher microbial biomass stock. However, when accounting also for the prokaryotic cells continuously transported out of the microcosms hardly any difference in the overall biomass production was revealed. Important in this context, differences in the rather constant numbers of sediment attached bacteria do not inform comprehensively about actual carbon conversion and bacterial growth rates. Similar results were obtained by Grösbacher et al. (2018) in sediment column studies. In fact, while 10 times the organic carbon was mineralized in sediments supplied with soil leachate (DOM-5x) compared to sediments supplied with groundwater DOM (DOM-1x), only one tenth the amount of carbon was assimilated on a cell level.

### Metabolic Flexibility of Aquifer Microbial Communities in Carbon Use

Long-term exposure to mainly refractory organic matter can be seen as ecological force that selects for microbes more talented in degradation of complex organic molecules. It is thus expected that microbial communities in pristine groundwater and aquifers poor in ready degradable organic matter are better adapted for the degradation of refractory organic material.

To directly compare the physiological status in terms of community-level sole-carbon-source utilization (Garland and Mills, 1991), water samples at the outflow of DOM-1x and DOM-5x supplied sediments were collected and incubated for 27 days with a multitude of organic substrates. Although the carbon substrate plate method is discussed controversial (Konopka et al., 1998; Ritz, 2007), it can provide conclusive indication for an adaptive change within microbial communities supplied by different organic matter qualities. Our results revealed that the DOM-1x fed communities lagged behind the DOM-5x fed communities in degradation of most organic carbon compound classes tested, with exception of the polymers. The number of complex compounds (e.g., glycogen, inulin, Tween 40 and 80, and cyclodextrin) metabolized by the DOM-1x microbial community was always higher than with the DOM-5x community. Even after 27 days, the latter was not able to mineralize some of the polymeric compounds (α‐ and γ-cyclodextrin). Similar to other studies (e.g., Purkamo et al., 2018), carbohydrates and carboxylic acids were the first compounds mineralized by the groundwater communities, independent of DOC concentration and environmental conditions.

### Link Between DOM Diversity and Microbial Community Composition

There is conclusive evidence from studies in different aquatic environments that changes in organic matter supply in terms of quantity and quality steer shifts in microbial community composition (Shi et al., 1999; Baker et al., 2000; Findlay et al., 2003; Carlson et al., 2004; Judd et al., 2006; Kritzberg et al., 2006; Li et al., 2012). This applies in particular, if the altered DOM supply lasts for longer than just a couple of hours or days (Herzyk et al., 2014, 2017). Since in sedimentary aquifers more than 90% of the microbial biomass is associated with sediment surfaces (Alfreider et al., 1997; Griebler et al., 2002), the investigations focused on the composition of the bacterial communities attached to sediments. Our experiment with the amendment of soil leachate to groundwater DOM at two very different ratios (DOM-1 × 1:2.5 to 1:4; DOM-5 × 4.4:1 to 7.2:1) revealed some similar changes in composition of bacterial communities in DOM-1x and DOM-5x fed sediments. In fact, compared to the situation before DOM supply, the overall fraction of Proteobacteria decreased from more than 90% to below 70%. In both treatments, the earlier dominance by *Betaproteobacteria* was taken over by *Alphaproteobacteria*. Moreover, *Actinobacteria* and *Bacteriodetes* gained importance (Figure 8). The relative decline in Proteobacteria was accompanied by the emergence (>1% relative abundance) of *Planctomycetes*, *Acidobacteria*, and *Nitrospirae*. In comparison to the DOM-5x sediments, the *Actinobacteria* were considerably higher in the DOM-1x sediments. Also, *Firmicutes* only appeared in the DOM-1x sediments (Figure 8). Overall, after 171 days of experiment, the bacterial communities fed by a low (DOM-1x) and a moderate (DOM-5x) soil DOM supply showed a similarity of 88% (Jaccard’s). In consequence, the addition of high complexity DOM from soil leachate, at low or moderate concentration, did not stimulate the fast growth of individual generalist species, but more evenly led to an increase, with only a few exceptions, of the detectable richness and diversity in the sediment communities. Due to the comparable low sequence depth revealed, a higher taxonomic resolution was not exercised.

The dominating groups of bacteria found in our aquifer sediments were in accordance of what is known from other groundwater ecosystem studies. A dominance of Proteobacteria was regularly reported (Shi et al., 1999; Detmers et al., 2004; Hendrickx et al., 2005; Boyd et al., 2007; Griebler and Lueders, 2009; Flynn et al., 2012; Gregory et al., 2014; Sirisena et al., 2018). Besides Proteobacteria, *Actinobacteria*, *Firmicutes*, *Bacteriodetes*, and *Nitrospirae* have frequently been detected at relevant abundances (Hendrickx et al., 2005; Griebler and Lueders, 2009; Zhou et al., 2012; Navarro-Noya et al., 2013; Gregory et al., 2014; Nowak et al., 2017; Bellini et al., 2018; Savio et al., 2018; Smith et al., 2018).

A dominance of Proteobacteria with contributions from *Betaproteobacteria*, *Alphaproteobacteria*, and *Gammaproteobacteria* was also found in other comparable sediment studies (Li et al., 2013; Griebler et al., 2016). In a column study, Li et al. (2013) observed significant differences in microbial community composition between top sediments continuously supplied by water carrying low or moderate BDOC. With distance to the inlet, the differences vanished. Another study by the same authors revealed an increase in relative abundance of *Betaproteobacteria* with higher BDOC concentrations (Li et al., 2012). A lower BDOC was accompanied by a higher relative abundance of *Firmicutes*, *Planctomycetes*, and *Actinobacteria*, a pattern that mirrored our own findings.

We are only beginning to understand whether and how energy-diversity relationships known from macroecology apply to complex natural bacterial communities. In fact, there is a growing body of evidence that diversity-productivity relationships also rule microbial communities (Smith, 2007).

Experimental studies that supplied sediment microbial communities report a decrease of microbial diversity in terms of richness and Shannon-Wiener diversity with higher DOM feed (Li et al., 2012, 2013). A positive relationship between bacterial richness and bioavailable organic matter was observed in Arctic deep-sea sediments, hinting at a positive energy-diversity relationship in oligotrophic regions (Bienhold et al., 2012).

Our sequencing data revealed that after 171 days of feeding sediment communities with either low or moderate DOM, bacterial richness and diversity increased in areas close to the microcosms inlet, however, with the DOM-5x sediments establishing more diverse microbial communities (Supplementary Figure S9). With distance to the fresh bioavailable organic carbon source, bacterial richness and Shannon-Wiener diversity declined. The DOM-1x fed sediments, at about 1-m distance from the microcosm inlet, did not anymore show a difference in terms of richness and diversity to the natural aquifer sediment communities at the beginning of the experiment.

## Conclusion

Groundwater microbial communities do not react very fast to the sudden availability of biodegradable organic carbon from soil in terms of degradation activity and production. This may reflect their long-term exposure and adaptation to typically energy-poor conditions. We expect active growth for only a small fraction of the community and probably even within populations (see recent publication by Kundu et al., 2020), while the other part is in a kind of survival mode and dormancy. It then takes days to weeks for incoming DOM being degraded and even longer before organic matter turnover results in a pronounced microbial growth. However, once conditioned, only a few dozen centimeters of microbial active aquifer sand are sufficient to deplete the labile fraction of DOM passing through. Taking the irregular inputs of BDOM into consideration, shallow aquifers are assumed to have a low resilience. Lacking a highly active and responsive microbial community, oligotrophic aquifers are at high risk of contamination with organic chemicals. Surprising also, even when allowed to adapt to the long-term supply of energy in form of labile organic matter, secondary production does not scale up linearly. The more labile carbon available and oxidized, the less carbon is seemingly assimilated. Currently, we may only speculate that in such cases, cells may run into the limitation by a specific nutrient or biomass is kept low due to an increasing importance of bacteriophages and/or heterotrophic protozoa, issues that await a detailed investigation. Comparably important, physiological adaptation of microbes in oligotrophic groundwater ecosystems at a cellular and biochemical resolution will be target of future studies.

## Data Availability Statement

The datasets generated for this study can be found in the NCBI accession number: PRJNA648309.

## Author Contributions

RH and CG designed the experiments, RH conducted the experiments, and RH, JU, and NH analyzed the samples including statistics. RH and CG took lead in writing the manuscript with contributions from JU and NH. All authors contributed to the article and approved the submitted version.

### Conflict of Interest

The authors declare that the research was conducted in the absence of any commercial or financial relationships that could be construed as a potential conflict of interest.
