# Supplementary material for: Linkage Between Dissolved Organic Matter Transformation, Bacterial Carbon Production, and Diversity in a Shallow Oligotrophic Aquifer: Results From Flow-Through Sediment Microcosm Experiments
Source: Front Microbiol. 2020 Nov 5;11:543567. doi: 10.3389/fmicb.2020.543567 (PMC7674671; doi:10.3389/fmicb.2020.543567)
Supplement: Supplementary file 1 [file Presentation_1.pdf]

Linkage Between Dissolved Organic Matter Transformation, Bacterial Carbon Production, and Diversity in a Shallow Oligotrophic Aquifer: Results from Flow-Through Sediment Microcosm Experiments

Roland Hofmann[1], Jenny Uhl[2], Norbert Hertkorn[2] and Christian Griebler,[1,3*]

Supplementary information

[1] Institute of Groundwater Ecology, Helmholtz Center Munich, Neuherberg, Germany

[2] Research Unit Analytical Biogeochemistry, Helmholtz Center Munich, Neuherberg, Germany

[3] Division of Limnology, Department of Functional and Evolutionary Ecology, University of Vienna, Vienna, Austria

Running Head: DOM Transformation in Oligotrophic Aquifers

*Correspondence: christian.griebler@univie.ac.at

Details on compositional changes of DOM with sediment passage

High-field NMR spectroscopy provides the capability for quantitative and non-destructive *de novo* determination of atom-specific chemical environments (describes chemical bonding to neighboring atoms) of polydisperse and molecularly heterogeneous DOM (Hertkorn et al., 2007; Powers et al., 2019). While NMR allows analysis and quantification of carbon- and hydrogen-based aliphatic, oxygenated and aromatic chemical environments of atoms, FT-ICR mass spectrometry enables to assign thousands of CHO, CHOS, CHNO and CHNOS molecular formulas directly out of complex mixtures (Koch et al., 2005; Hertkorn et al., 2008; Kujawinski et al., 2009). FT-ICR mass spectrometry of DOM is however subject to ionization selectivity, which regularly causes mass peak intensities to deviate from actual molecular concentrations (Hertkorn et al., 2008). Moreover, many (several thousands and more) isomeric molecules may project on any individual mass peaks, further obstructing direct relationships between mass spectra and actual defined individual molecules. Because NMR and FT-ICR mass spectra each provide valuable but rather complementary information about structure and molecular composition of DOM (Hertkorn et al., 2007), both technologies were applied.

^{1}H NMR spectra served to constrain compositional changes of DOC in selected samples collected from non-amended groundwater and the DOM-5x approach. The ^{1}H NMR spectra of groundwater DOM and the feeding solution DOM-5x were largely dissimilar as expected (Figure 4 in the manuscript). Groundwater DOM featured the highest content of pure aliphatics (CCC**H** units; Table 1) and the lowest content of oxygenated aliphatic groups and protons bound to unsaturated carbon (OC**H** and C_{sp2}**H** units; Table 1), with olefinic protons particularly depleted. In contrast, feeding solution DOM-5x was much more oxidized than the groundwater DOM sample and contained large fractions of potentially lignin-derived aromatic and phenolic units but only minor proportions of olefins (Figure S1). The group of oxygenated aliphatics

included a large complement of carbohydrates, aromatic and aliphatic methyl esters and methyl ethers, peptides and carboxyl-rich alicyclic matter (CRAM) (Figure 4 in the manuscript).

The small compositional changes between the pure groundwater infiltrated into the microcosm before any feeding solution was supplied and the outflow at t0 (Table 1 in the manuscript) most probably reflected slight attenuation of DOM due to adsorption to the sediment as well as some production of limited amounts of microbial metabolites in a nearly equilibrated system. These showed sharp NMR resonances in all sections of the ^{1}H NMR spectrum (Figure S1-C & S1-D), including $C_{ar}\underline{\mathbf{H}}$, $OC\underline{\mathbf{H}}_n$ and $CC C\underline{\mathbf{H}}$ derived aliphatic metabolites, likely including derivatives of short-chain fatty acids (δ_H ~ 1.4-0.9 ppm).

The DOM-5x feeding solution at the inflow and the DOM in the outflow at t7 were overall comparable in their structural characteristics (Figure S1-C & S1-D), underlining a delayed onset of microbial transformation of soil leachate DOM added to groundwater. However, the DOM from the microcosm outlet at t7, already showed a selective depletion in aromatic methylesters and methyl ethers by ~35% (Figure S1-D), in line with a known preferential sorption of aromatic compounds. Besides sorption being involved in DOM attenuation, indication of microbial processing of DOM came from the presence of different sharp NMR resonances in eluates from t7 and t171, which probably originated from a few but rather abundant microbial metabolites. Compared with groundwater inflow, pure and functionalized aliphatics were found relatively enriched at t7. In particular, aliphatics became likely more branched upon processing of DOM as shown by the overall broadened NMR resonances at δ_C ~ 0.8-1.7 ppm (Figure S1-B). A higher abundance of CRAM molecules (carboxyl-rich alicyclic molecules; Hertkorn 2006), pointed at a higher overall extent of carboxylation, and depletion of certain oxygenated molecules (δ_H > 4 ppm) at t7 (Figure S1-A & S1-B) implied early preferential processing of DOM molecules with higher proportions of oxygen-containing

functional groups. After 171 days of experiment, DOM samples at the outflow of the DOM-5x fed sediment zones were found very similar in composition to the DOM in the natural groundwater. The long-term supply of DOM-5x thus led to an altered dynamic equilibrium, with increased proportions of aromatic and olefinic molecules as well as aliphatic methyl esters, compared with the DOM from t0 (Figure S1-A & S1-B). In fact, initial sorption of these compounds later dropped out and these compounds quantitatively passed the sediments.

The high sensitivity, excellent mass resolution and mass accuracy of FT-ICR mass spectrometry allowed profiling of the temporal changes of organic matter for both the DOM-1x and the DOM-5x approach. Several thousands of mass peaks were assigned to CHO, CHNO, CHOS and CHNOS molecular formulas (Figure 5 in the manuscript; Table S1; Table S2). FT-ICR-MS spectra of groundwater as well as all samples collected at the microcosm outlets showed the skewed, near Gaussian distribution of mass peaks typical of common organic matter, with mass differences dominated by the most abundant ±DBE (Δm = 2.1057 Da) and $\pm CH_2$ (Δm = 14.0156 Da) groups (Figure 5 in the manuscript). Feeding solution DOM-5x showed a higher mass range and a more complex pattern than the other SPE-DOM samples, in line with the prominent signature of oxygenated aliphatic and aromatic functional groups observed already in NMR spectra (Figure S1). In accordance with NMR results for the DOM-5x experiment, hierarchical cluster analysis (HCA) and principal component analysis (PCA) grouped the feeding solution DOM-5x and its eluate from t7 together, well separated from the other three DOM samples (Figure 4-A & 4-B in the manuscript). In the DOM-1x experiment, the PCA-based distinction of all eluates was less pronounced than with the DOM-5x approach that showed a more distinct temporal evolution (Figure 5-A in the manuscript; Figure S1-S3).

FT-ICR mass spectrometry revealed a different temporal evolution of DOM processing for CHO, CHNO, CHOS and CHNOS molecular series in DOM-1x and DOM-5x treatments

(Figure S4, Figure S5). Figures S4 and S5 display differences between mass spectra from samples at time points t0 [t0 *minus* [DOM-1(5)x + ground water)] , t7 and t171, and therefore refer to molecular compositions affected by degradation and processing of organic matter. The molecular diversity of organic molecules affected by degradation was, broadly spoken, higher in DOM-5x samples than in DOM-1x samples, because their van Krevelen and mass-edited H/C ratio diagrams occupied larger areas. The effects of feeding solution on the composition of outflow waters were more distinct in case of the DOM-5x experiment (Figure S5). While the DOM-1x experiments showed lesser variations between t0 and t7 samples and even smaller ones between t171 and natural groundwater DOM (Figure S4), the respective changes in the DOM-5x experiment showed a distinct processing of DOM.

General FT-ICR-MS-derived bulk characteristics, like averaged elemental ratios and relative unsaturation as expressed by DBE/C varied only slightly across all samples (Table S1; Table S2) reflecting the overall gross similarity of eluates and effects of massive projection of structural isomers onto common molecular compositions (Hertkorn et al., 2007) and ionization selectivity out of the very complex mixture of DOM molecules (Hertkorn et al., 2008). The temporal evolution of the average mass in DOM-5x experiments was consistent with a continual degradation from larger to smaller molecules (Table S2). Comparative analysis of CHO, CHNO, CHOS and CHNOS molecular series of individual samples in comparison with groundwater inflow revealed significant information about the temporal evolution and selective processing of DOC molecules (Figure S4; Figure S5). Overall, the molecular selectivity of processing of CHO and CHNO compounds appeared similar, whereas processed less numerous CHOS compounds were in general more depleted in oxygen and of lower mass. Processing of the very numerous CHNOS compounds was clearly distinct as described below, Turnover of CHO compounds between t0 and t7 moved towards smaller molecules and slightly more

deoxygenation at otherwise similar H/C ratios (H/C ratio ~ 0.8-1.5). At t171, the group of oxygenated, lignin-like compounds of relatively high mass (m/z > 450 Da) was depleted (Figure S4-F, Figure S5-F). Furthermore, only highly oxygenated CHO (with m/z < 450) and CHNO compounds (O/C ratio > 0.4) disappeared. Selectively processed CHOS molecules were lipid-like and of weak to considerable unsaturation.

Overall, the FT-ICR-MS derived molecular selectivity of processing of CHO and CHNO compounds appeared grossly similar, however, processed CHNO compounds were less numerous than CHO molecules and also chemically less diverse as seen by the smaller space occupied in the van Krevelen diagrams (Figure S4, Figure S5). The temporal evolution of the less numerous CHOS compounds primarily involved aliphatic compounds with a wide range of oxygenation, comprising common DOM molecules as well as sulfolipid-like compounds.

At t7, the proportions of processed DOM molecules had increased in comparison, whereas lipid-like compounds and less oxidized CHOS compounds were transformed only later (t171), indicating a slow progressive defunctionalization toward more saturated and less oxygenated compounds (Figure S4, Figure S5). CHOS compounds were in general more depleted in oxygen and of lower mass for both DOM-5x and DOM-1x treatments. This is remarkable, because common sulfur functional groups in DOM will most probably carry several oxygen atoms (e.g. $–OSO_3H$ and $–OSO_2H$ groups) under oxic conditions. Hence, the parent molecules of selectively reacted CHOS compounds were rather oxygen-depleted and of considerably lower mass compared with their CHO counterparts (Figure S4-E, Figure S5-E). Processing of the very numerous CHNOS compounds throughout the 171 days, which showed a wide range of relative unsaturation, was clearly different from those of CHNO and CHOS compounds for both, DOM-1x and DOM-5x treatments (CHOS and CHNOS compounds were confirmed in the mass spectra by their conspicuous doublet structure $\Delta m = C_{-3}H_4S = 2.4$ mDa; Schmitt-

Kopplin et al., 2010). Processed CHNOS compounds comprised otherwise rarely observed clusters of oxygen-deficient (O/C ratio < 0.5) aromatic compounds and another set of highly oxygenated (O/C ratio > 0.5) compounds of considerable aliphaticity (H/C ratio ~ 1.4 – 2.3), some of which might represent substituted carbohydrates (e.g. microbial metabolites). The latter group was particularly abundant in DOM-5x in which the most unsaturated, i.e. aromatic CHNOS compounds (H/C ratio ~ 0.75) showed a distinct cluster of high mass molecules (m/z > 500), suggesting different structures as well (Figure S5-F).

When passing through the sediments, CHO and CHNO molecules of average H/C and relatively high O/C ratios in the groundwater have been selectively attenuated at t0 (previous to DOM supply) in experiment DOM-5x, suggesting initial degradation or selective adsorption of highly functionalized DOM molecules (Figure S5-A). At t7 (Figure S5-B), the profile of processed CHO and CHNO molecules had shifted toward highly oxidized lignin- (O/C ratio > 0.5) and tannin-like (O/C ratio > 0.7) compounds of high mass, in line with the appearance of forest-soil derived compounds in the ^{1}H NMR spectra (Figure S1). Preferential processing of CHOS compounds shifted from common DOM molecules and marginally unsaturated sulfolipid-like compounds at t0 to a much more restricted set of less oxidized molecules at t7, whereas at t171 more unsaturated CHOS compounds of substantial overall chemical diversity have been selectively transformed (Figure S5-A-C). At t0, a very few CHNOS compounds of average H/C and O/C ratios and a large set of oxygen-depleted (O/C ratio < 0.4) molecules with a wide-ranging diversity of relative unsaturation from aromatic to near fully saturated (H/C ratio ~ 0.8-1.9) molecules with m/z < 500 Da have been selectively transformed (Table S2, Figure S5-D). A few aliphatic and oxidized CHNOS compounds had also been depleted. At t7 and t171, two very distinctive major sets of CHNOS compounds, one compact but molecularly widely diverse set of minimal to considerable oxygenation (O/C ratio ~ 0.05-0.5)

and aromatic to nearly fully saturated (H/C ratio ~ 0.6-1.85), and a similarly diverse but less numerous assembly of oxygenated (O/C ratio ~ 0.4-0.9) and rather aliphatic (H/C ratio ~ 1.1-2.2) CHNOS compounds of relatively smaller mass (m/z < 650 Da) have been substantially depleted (Figure S5-E & Figure S5-F).

The temporal evolution of DOM-1x was less distinct than that in DOM-5x experiments (Figure S4). In particular, turnover of CHO compounds between t0 and t7 moved towards smaller molecules and slightly more deoxygenation at otherwise similar H/C ratios (H/C ratio ~ 0.8-1.5); at t171 a limited set of oxygenated, lignin-like compounds of relatively high mass (m/z > 450 Da) was depleted. At t171, only highly oxygenated CHO (with m/z < 450) and CHNO compounds (O/C ratio > 0.4) had been depleted, reflecting a greater relative stability of these highly oxidized compounds when compared with common DOM/CRAM molecules of more average H/C and O/C ratios (Figure S4-C & Figure S4-F). Selectively processed CHOS molecules were lipid-like and of weak to considerable unsaturation.

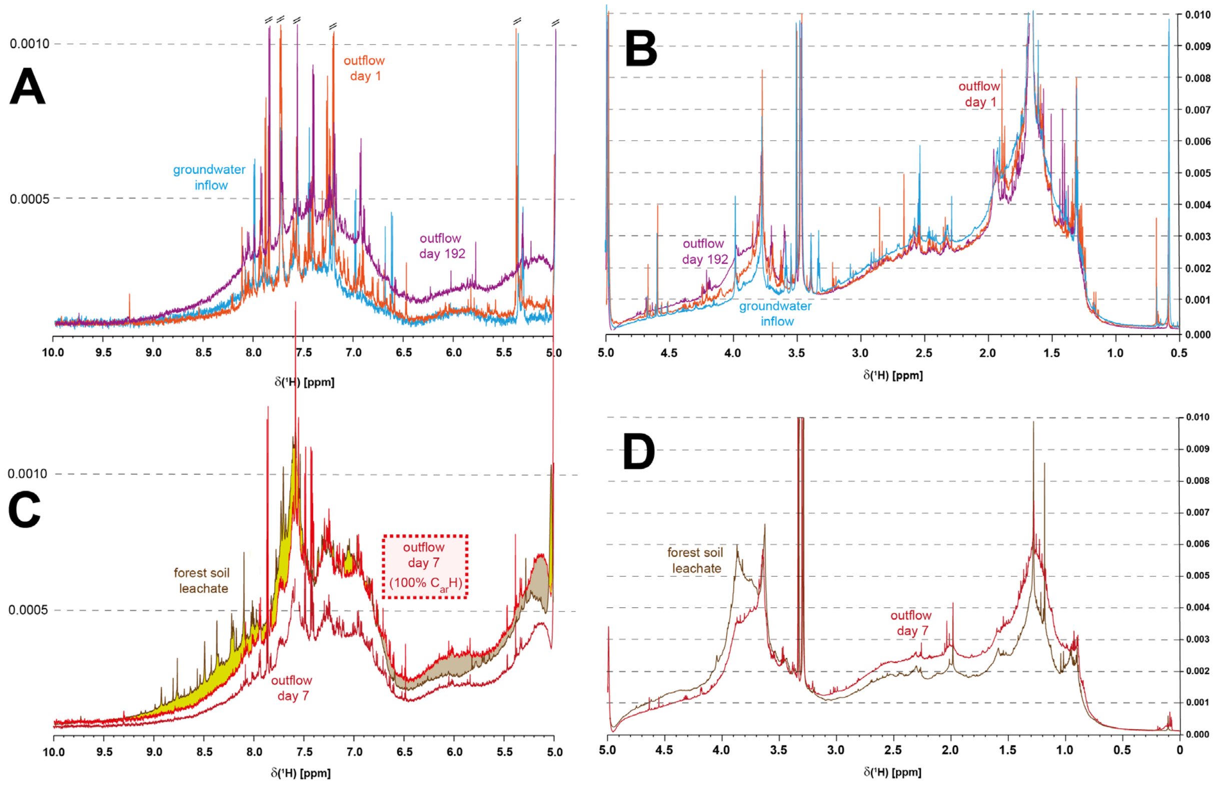

Figure S1. Overlay of area-normalized ^{1}H NMR spectra (800 MHz, CD_3OD) for groundwater, feeding solution DOM-5x and DOM samples from the DOM-5x zone outflow at different time points showing C_{sp2}**H** (panels A, C) and C_{sp3}**H** (panels B, D) chemical environments. Panel C shows at first area normalized spectra within the entire range of chemical shift (δ_H: 0.5-10 ppm) which primarily depict relative depletion of C_{sp2}**H** based chemical environments across the board for outflow sample day 7. Area normalized spectra computed solely within the zone of C_{sp2}**H** units (δ_H: 5-10 ppm) more instructively visualize structural variations resulting from microbial processing, and the difference between the two latter spectra is highlighted in light brown (outflow day 7 *minus* forest soil leachate) and light green (forest soil leachate *minus* outflow day 7) for improved contrast. Forest soil leachate displays higher proportions of polycarboxylated benzene derivatives and polyaromatic compounds (δ_H >7.6 ppm) whereas outflow at day 7 exhibits relatively higher proportions of olefins, likely indicative of natural products, lignins and oxygenated aromatic molecules (δ_H ~ 5.1-6.7 ppm).

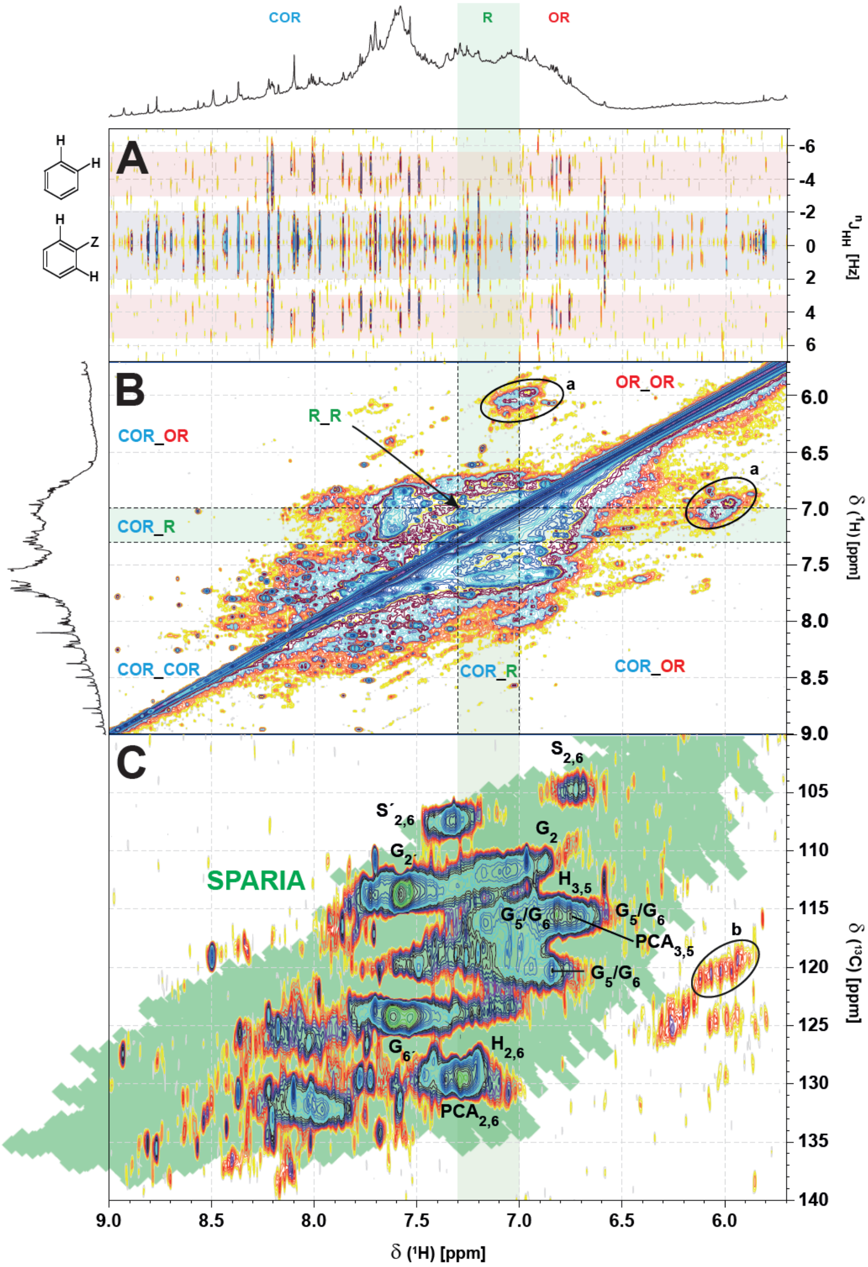

Figure S2. 2D NMR spectra of forest soil leachate, section of $C_{sp2}H$ units (panel A): ^{1}H, ^{1}H JRES NMR spectrum shows near absence of trans-double bonds and abundant ortho- and meta-protonated aromatic molecules; (panel B) ^{1}H, ^{1}H TOCSY NMR spectrum shows dominance of $^{3/4}J$-couplings between carboxylated aromatic molecules (COR), indicating multiple carboxylation; couplings between COR (electron withdrawing substituents; $\delta_H > 7.3$ ppm), R (neutral substituents H, R_{alkyl}; green shade, δ_H: 7.0 – 7.3 ppm), and OR (electron-donating substituents; ; δ_H: 6.5 – 7.0 ppm) substituents also occur, testifying to a substantial diversity of chemical environments. Section a: minor set of polarized double bonds (–C**H**=C**H**-C=O units) and (panel C) ^{1}H, ^{13}C HSQC NMR spectrum indicates major contributions of lignins (annotation equals that in ref. Kim and Ralph, 2010); the green section indicates the chemical shift range accessible by single aromatic rings; the numerous cross peaks at $\delta_H > 7.3$ ppm indicate abundance of (poly)carboxylated molecules (SPARIA: substitution patterns in aromatic rings by increment analysis; Perdue et al., 2007); section b: polarized double bonds, with other HSQC cross peaks originating from double bonds in vicinity.

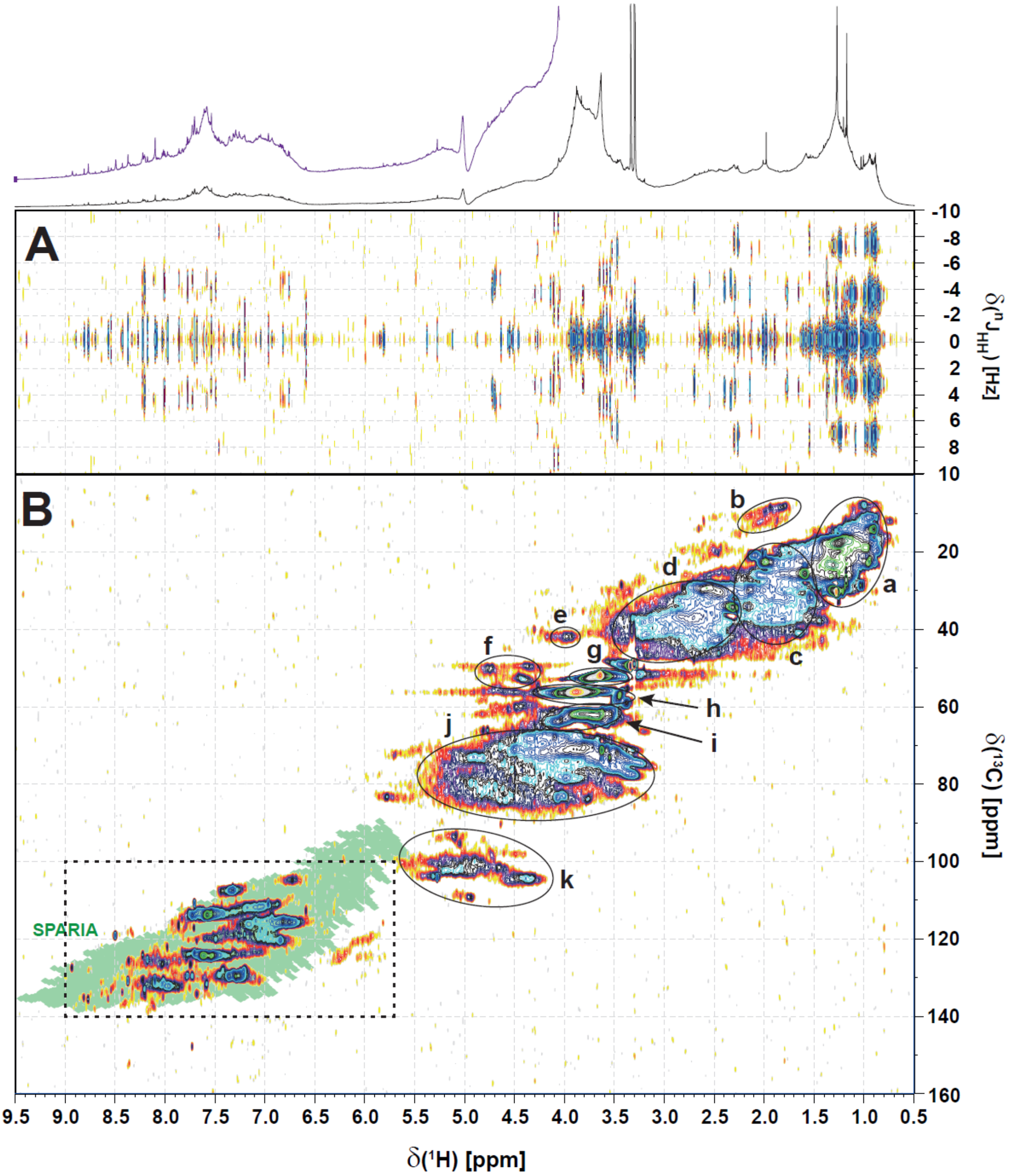

Figure S3. 2D NMR spectra of forest soil leachate (panel A): ^{1}H, ^{1}H JRES NMR spectrum; (panel B) ^{1}H, ^{13}C HSQC NMR spectrum: section a: C-$\underline{\mathbf{CH}}_3$ cross peaks; section b: C=C-$\underline{\mathbf{CH}}_3$ and -S$\underline{\mathbf{CH}_3}$ cross peaks; section c: -$\underline{\mathbf{CH}_n}$-$CH_n$-COOH cross peaks (n: 1, 2); section d: -C-$\underline{\mathbf{CH}_n}$-COOH cross peaks (n: 1, 2); section e: -CONH-$\underline{\mathbf{CH}_2}$-glycine in peptides; section f: CONH-$\underline{\mathbf{CH}_n}$- CHα in peptides (amino acids other than glycine); section g: methoxy (O$\underline{\mathbf{CH}_3}$) cross peaks: aliphatic and aromatic methyl esters $\underline{\mathbf{H_3C}}$O-C(=O)-C-; section h: aromatic and aliphatic methyl ethers $\underline{\mathbf{H_3C}}$O-C-C; section i: oxomethylene (O$\underline{\mathbf{CH}_2}$) cross peaks, likely from carbohydrates; section j: $OC_2$$\underline{\mathbf{CH}}$ cross peaks, carbohxydrates, ethers, esters, oxidation products of carotenoids; section k: O_2**CH** anomeric cross peaks from carbohydrates; dotted box cf. Figure S2 (**$C_{sp2}H$** units).

Table S1. Counts of common mass peaks of SPE-DOM (DOM-1x feeding solution) as computed from negative electrospray ionization (ESI) 12T FT-ICR mass spectra for singly charged ions.

Samples DOM-1x	groundwater inflow	forest soil leachate	t0	t7	t171
total counts of assigned mass peaks	6925	5241	6970	6756	7182
CHO compounds	2097 (30%)	1935 (37%)	2186 (31%)	2136 (32%)	2229 (31%)
CHOS compounds	1039 (15%)	648 (12%)	900 (13%)	880 (13%)	947 (13%)
CHNO compounds	1953 (28%)	1639 (31%)	2012 (29%)	2025 (30%)	2105 (29%)
CHNOS compounds	1836 (27%)	1019 (19%)	1872 (25%)	1715 (25%)	1901 (26%)
average H [%]	44.2	45.8	44.1	44.2	44.0
average C [%]	37.2	37.5	37.2	37.1	37.4
average O [%]	14.9	13.8	15.0	14.9	14.8
average N [%]	2.2	1.8	2.2	2.3	2.3
average S [%]	1.5	1.1	1.5	1.5	1.5
computed average H/C ratio	1.06	1.12	1.06	1.07	1.06
computed average O/C ratio	0.37	0.36	0.37	0.38	0.37
computed average C/N ratio	11.7	12.4	11.5	11.4	11.5
computed average C/S ratio	13.5	15.5	13.2	12.8	12.9
average carbon oxidation state of CHO compounds	-0.32	-0.40	-0.32	-0.31	-0.32
average DBE	9.3	9.0	9.3	8.9	9.2
average DBE/C	0.4	0.4	0.5	0.5	0.5
mass weight average [Da]	439.8	422.4	432.9	420.2	426.5

Table S2. Counts of common mass peaks of SPE-DOM (DOM-5x feeding solution) as computed from negative electrospray ionization (ESI) 12T FT-ICR mass spectra for singly charged ions

Samples DOM-5x	groundwater inflow	forest soil leachate	t0	t7	t171
total counts of assigned mass peaks	7709	7541	7096	7774	7473
CHO compounds	2883 (37%)	3153 (42%)	2239 (32%)	2834 (36%)	2572 (34%)
CHOS compounds	655 (8%)	481 (6%)	991 (14%)	791 (10%)	918 (12%)
CHNO compounds	1807 (23%)	1773 (24%)	2036 (29%)	1928 (25%)	2244 (30%)
CHNOS compounds	2364 (31%)	2134 (28%)	1830 (26%)	2221 (29%)	1739 (23%)
average H [%]	41.6	41.7	44.3	41.6	44.0
average C [%]	37.4	37.6	37.2	38.1	37.4
average O [%]	17.3	17.7	14.8	16.8	14.8
average N [%]	2.1	1.9	2.2	2.1	2.3
average S [%]	1.6	1.2	1.5	1.4	1.5
computed average H/C ratio	1.01	1.02	1.07	1.05	1.06
computed average O/C ratio	0.42	0.43	0.37	0.41	0.37
computed average C/N ratio	11.9	11.9	11.7	12.2	11.5
computed average C/S ratio	13.6	15.2	14.0	15.2	12.9
average carbon oxidation state of CHO compounds	-0.17	-0.16	-0.33	-0.23	-0.32
average DBE	10.6	10.9	9.3	10.6	9.15
average DBE/C	0.5	0.5	0.4	0.5	0.45
mass weight average [Da]	482.9	492.8	438.4	479.6	426.5

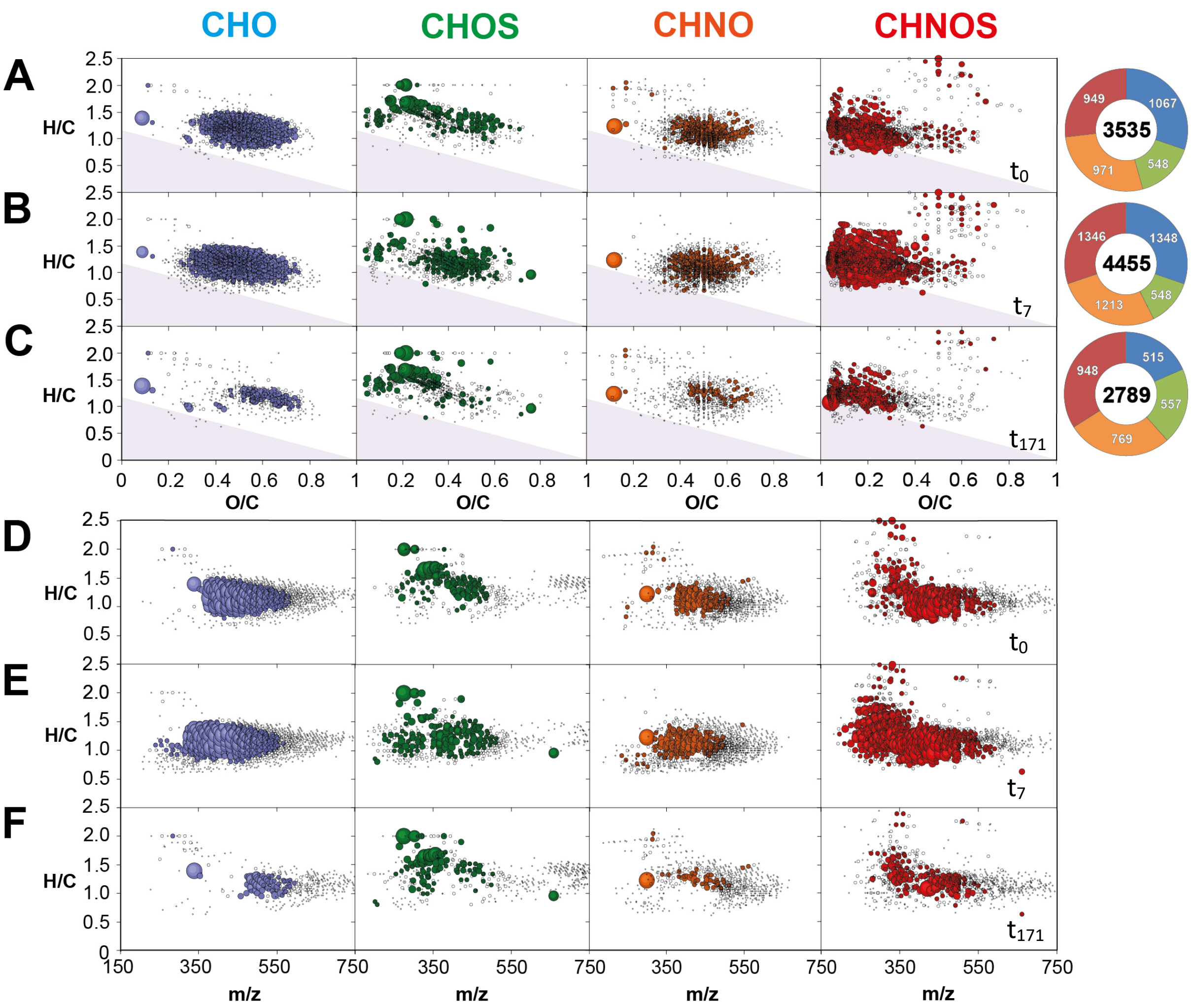

Figure S4. Van Krevelen diagrams (panels A, B, C) and mass-edited H/C rations (panels D, E, F) of DOM-1x experiment, derived from (-)ESI FT-ICR mass spectra, with distinct CHO (blue), CHOS (green), CHNO (orange) and CHNOS (red) molecular series and counts indicated by ring charts. The plotted data reflect *difference spectra*, i.e. t0 minus [DOM-1(5)x + ground water at ti], which were computed to emphasize DOM processing of groundwater inflow SPE-DOM and show CHO, CHOS, CHNO and CHNOS compounds, which were *declining* throughout the experiment (cf. text). Bubble areas represent relative mass peak amplitudes. The purple triangle indicates presence of aromatic molecules with aromaticity index AI > 0.5 (Koch and Dittmar, 2006).

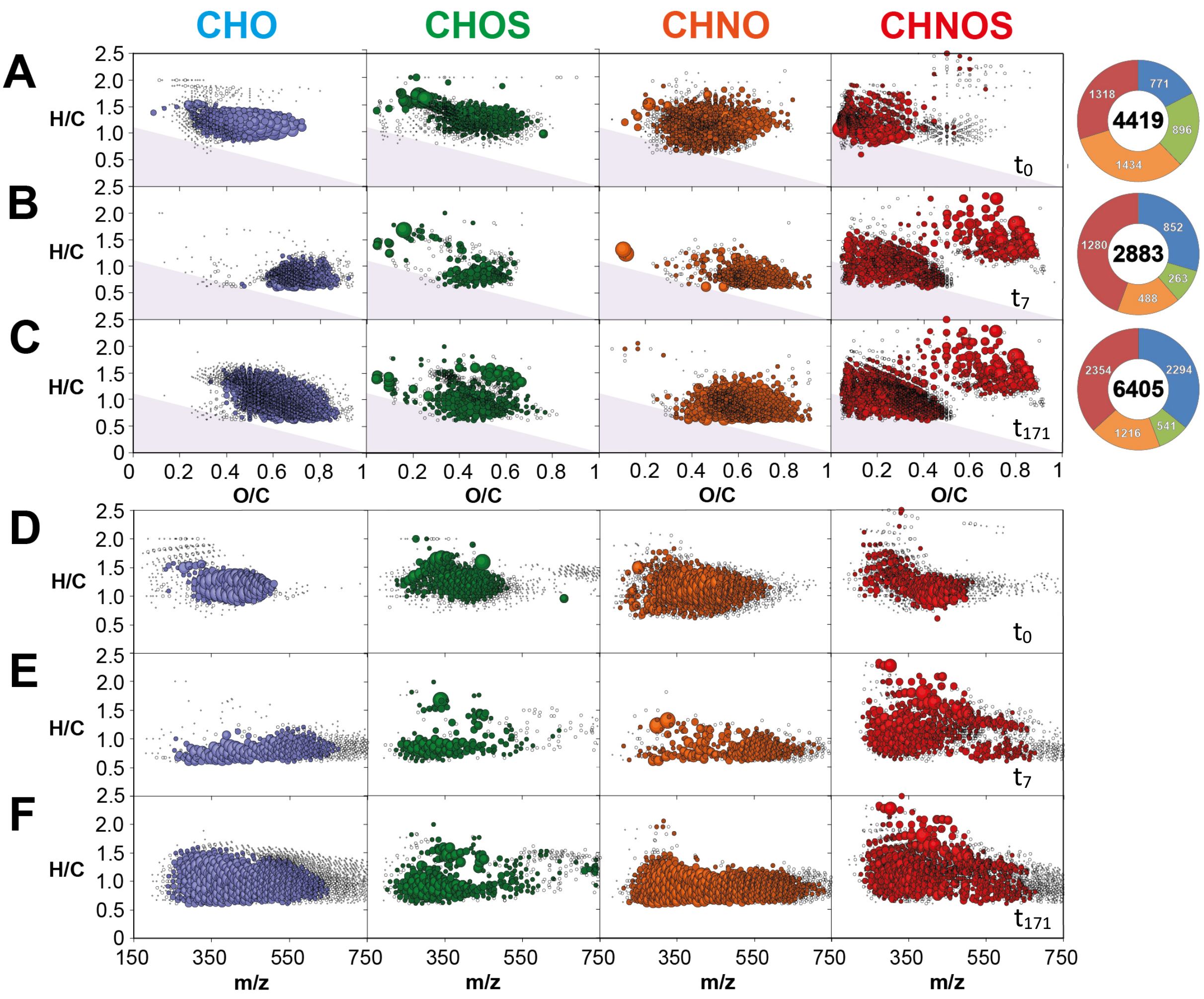

Figure S5. Van Krevelen diagrams (panels A, B, C) and mass-edited H/C rations (panels D, E, F) of DOM-5x experiment, derived from (-)ESI FT-ICR mass spectra, with distinct CHO (blue), CHOS (green), CHNO (orange) and CHNOS (red) molecular series and counts indicated by ring diagrams. The plotted data reflect *difference spectra*, i.e. t0 minus [DOM-1(5)x + ground water at ti], which were computed to emphasize DOM processing of groundwater inflow SPE-DOM and show CHO, CHOS, CHNO and CHNOS compounds, which were *declining* throughout the experiment (cf. text). Bubble areas represent relative mass peak amplitudes. The purple triangle indicates presence of aromatic molecules with aromaticity index AI > 0.5 (Koch and Dittmar, 2006).

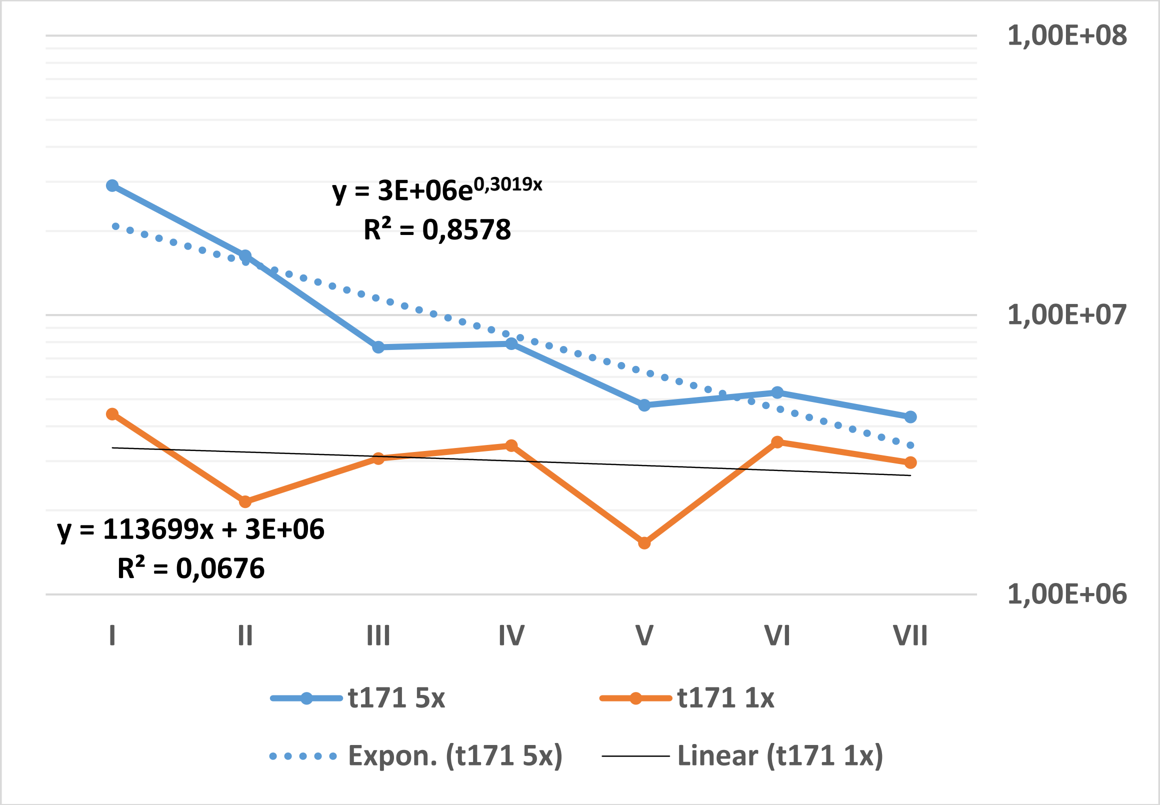

Figure S6. Inlet to outlet gradient of sediment bacterial biomass at the end of the experiment in DOX-1x (orange) and DOM-5x (blue) fed sediments. The DOM-5x fed sediment exhibit (best fit) an exponential decrease in cell numbers from inlet to outlet. With the DOM-1x fed sediment, there is no clear trend and no significant relationship. However, the pattern can be described by a linear decrease in cell number from the microcosm's inlet to outlet.

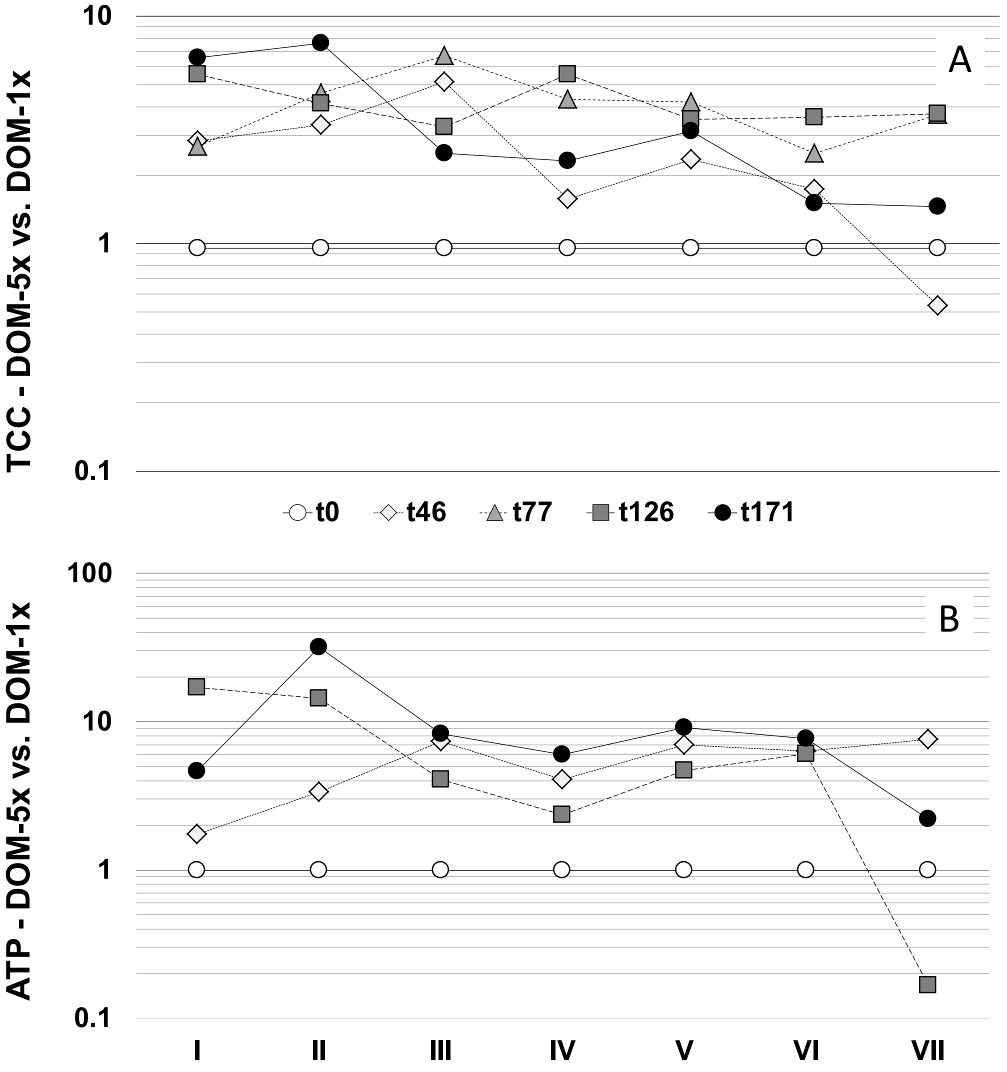

Figure S7. Ratio for TCC (A) and ATP values (B) when comparing sediments receiving DOM-5x and DOM-1x feeding solution for individual time points and with distance to the microcosms inlet.

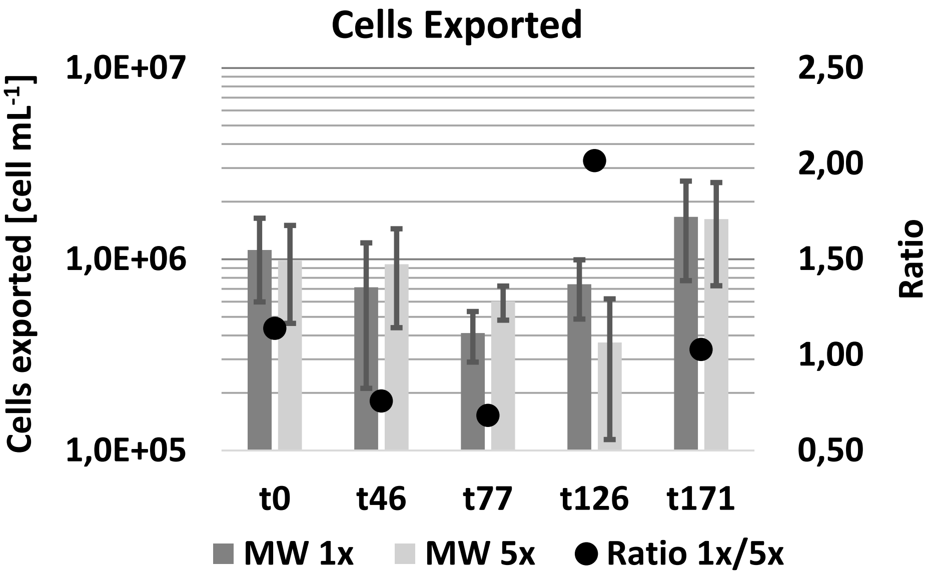

Figure S8. Concentration of bacterial cells transported out of the different treatment zones of the microcosms, i.e. DOM-1x and DOM-5x fed sediments, and the ratio of cell concentrations in the outflow water of DOX-1x and DOM-5x treated sediment segments. Values are means ± SD.

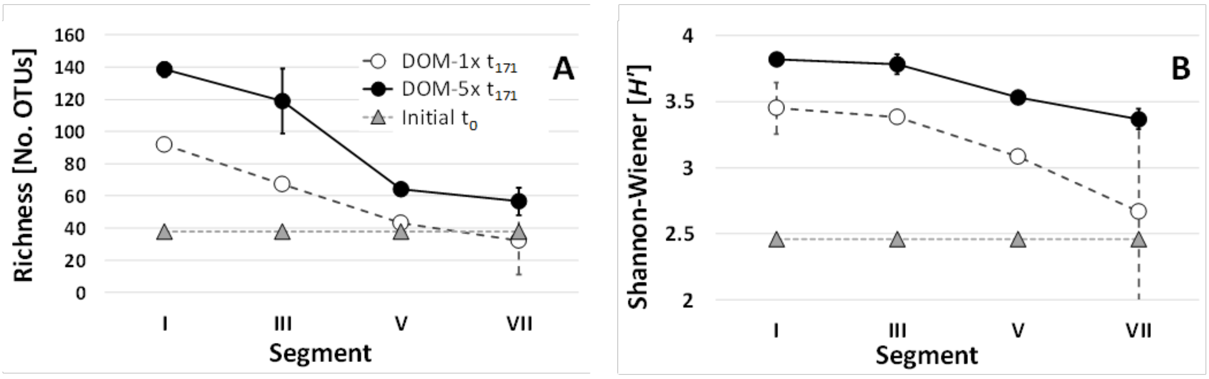

Figure S9. Bacterial richness (A) and Shannon-Wiener diversity (B) in fresh aquifer sediment and in the sediments receiving DOM-1x and DOM-5x at day 171.

References

Hertkorn N, Frommberger M, Witt M, Koch BP, Schmitt-Kopplin P & Perdue EM (2008) Natural organic matter and the event horizon of mass spectrometry. *Anal Chem* **80:** 8908-8919.

Hertkorn N, Ruecker C, Meringer M, Gugisch R, Frommberger M, Perdue EM, Witt M & Schmitt-Kopplin P (2007) High-precision frequency measurements: indispensable tools at the core of the molecular-level analysis of complex systems. *Anal Bioanal Chem* **389:** 1311-1327.

Kim H & Ralph J (2010) Solution-state 2D NMR of ball-milled plant cell wall gels in DMSO-d6/pyridine-d5. *Org Biomol Chem* **8:** 576-591.

Koch BP, Witt MR, Engbrodt R, Dittmar T, & Kattner G (2005) Molecular formulae of marine and terrigenous dissolved organic matter detected by electrospray ionization Fourier transform ion cyclotron resonance mass spectrometry. *Geochim Cosmochim Acta* **69:** 3299-3308.

Koch BP & Dittmar T (2006) From mass to structure: An aromaticity index for high-resolution mass data of natural organic matter. *Rapid Comm Mass Spect* **20:** 926-932.

Kujawinski EB, Longnecker K, Blough NV, Del Vecchio R, Finlay L, Kitner JB & Giovannoni SJ (2009) Identification of possible source markers in marine dissolved organic matter using ultrahigh resolution mass spectrometry. *Geochim Cosmochim Acta* **73:** 4384-4399.

Perdue EM, Hertkorn N & Kettrup A (2007) Substitution patterns in aromatic rings by increment analysis. Model development and application to natural organic matter. *Anal Chem* **79:** 1010-1021.

Powers, LC, Hertkorn, N, McDonald, N, Schmitt-Kopplin, P, Del Vecchio, R, Blough, NV & Gonsior, M (2019) Sargassum sp. Act as a Large Regional Source of Marine Dissolved Organic Carbon and Polyphenols. *Global Biogeochem Cycles* **33:** 1423-1439.

Schmitt-Kopplin P, Gelencsér A, Dabek-Zlotorzynska E, Kiss G, Hertkorn N, Harir M, Hong Y & Gebefüge I (2010) Analysis of the unresolved organic fraction in atmospheric aerosols with ultrahigh-resolution mass spectrometry and nuclear magnetic resonance spectroscopy: organosulfates as photochemical smog constituents. *Anal Chem* **82:** 8017-8026.
